# Solar powered biohydrogen production requires specific localization of the hydrogenase†
†Electronic supplementary information (ESI) available: Supplementary Fig. 1–12 and supplementary Table 1. See DOI: 10.1039/c4ee02502d



**DOI:** 10.1039/c4ee02502d

**Published:** 2014-09-23

**Authors:** Nigel J. Burroughs, Marko Boehm, Carrie Eckert, Giulia Mastroianni, Edward M. Spence, Jianfeng Yu, Peter J. Nixon, Jens Appel, Conrad W. Mullineaux, Samantha J. Bryan

**Affiliations:** a Systems Biology Centre , Coventry House , University of Warwick , Coventry , CV4 7AL , UK; b Imperial College London , South Kensington Campus , London , SW7 2AZ , UK; c Biosciences Centre , National Renewable Energy Laboratory , Golden , Colorado 80401 , USA; d Renewable and Sustainable Energy Institute, University of Colorado Boulder , Boulder , CO 80309 , USA; e School of Biological and Chemical Sciences , Queen Mary University of London , Mile End Road , London , E1 4NS , UK . Email: samantha_bryan@hotmail.co.uk; f Pharmaceutical Science Division , King's College London , Franklin-Wilkins Building, 150 Stamford Street , London , SE1 9NH , UK; g Botanical Institute , University of Kiel , Am Botanischen Garten 1-9 , 24118 Kiel , Germany

## Abstract

Cyanobacteria contain a bidirectional [NiFe] hydrogenase which transiently produces hydrogen upon exposure of anoxic cells to light, potentially acting as a “valve” releasing excess electrons from the electron transport chain. However, its interaction with the photosynthetic electron transport chain remains unclear. By GFP-tagging the HoxF diaphorase subunit we show that the hydrogenase is thylakoid associated, comprising a population dispersed uniformly through the thylakoids and a subpopulation localized to discrete puncta in the distal thylakoid. Thylakoid localisation of both the HoxH and HoxY hydrogenase subunits is confirmed by immunogold electron microscopy. The diaphorase HoxE subunit is essential for recruitment to the dispersed thylakoid population, potentially anchoring the hydrogenase to the membrane, but aggregation to puncta occurs through a distinct HoxE-independent mechanism. Membrane association does not require NDH-1. Localization is dynamic on a scale of minutes, with anoxia and high light inducing a significant redistribution between these populations in favour of puncta. Since HoxE is essential for access to its electron donor, electron supply to the hydrogenase depends on a physiologically controlled localization, potentially offering a new avenue to enhance photosynthetic hydrogen production by exploiting localization/aggregation signals.

## Abstract 2

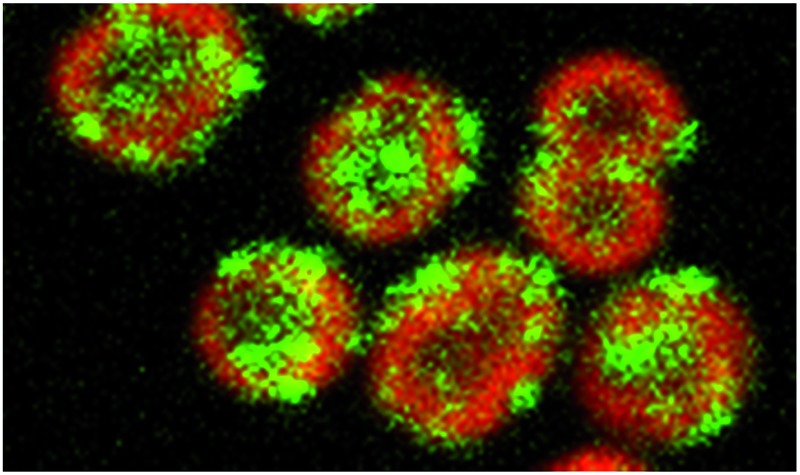
Subcellular localization of the cyanobacterial hydrogenase is under physiological control and is crucial for photosynthetic hydrogen production.

Broader context

Hydrogen has real potential as a clean renewable fuel, producing water on combustion. Solar powered bio-hydrogen has several advantages; it is relatively harmless to the organisms producing it and is easily separated from the growth media. Photoautotrophic microbes like cyanobacteria can utilize cheap and plentiful sources of carbon and electrons for growth and hydrogen production making them self-sustaining production vehicles. However, diverting a high proportion of reducing power to hydrogen production poses significant challenges, exacerbated considerably by uncertainties in how the hydrogenase interacts with the electron transport chain. We investigated hydrogenase location and behaviour in the model unicellular cyanobacterium *Synechocystis* sp. PCC 6803, location having a direct bearing on access to electron donors. We demonstrate that the hydrogenase has two distinct physiologically-controlled localization mechanisms that partition it within the thylakoids, either dispersed uniformly through the thylakoids or aggregated into discrete puncta. Crucially, electron supply and hydrogen production depend on localization. Determination of the molecular basis for control of hydrogenase location could thus pave the way to engineering improved cyanobacterial cells for solar-powered bio-hydrogen production.

## Introduction

Cyanobacteria are photoautotrophic microorganisms that can utilize cheap and plentiful sources of carbon, electrons, and energy for growth. They possess hydrogenases capable of combining electrons originally derived from water with protons to produce H_2_ under specific conditions.^1^ Therefore diverting reducing power to H_2_ production from the photosystems could potentially be an energetically efficient method of solar-powered biofuel production.^2^ However, in addition to the engineering challenges that this would pose, there are significant biological challenges. Photosynthetic H_2_ production in cyanobacteria has only been observed in transient bursts when dark-adapted, anoxic cells are first exposed to light;^3,4^ H_2_ production then ceases in parallel with the increase in O_2_ concentration due to the water-splitting activity of Photosystem II,^3–5^ all cyanobacterial hydrogenases being to some degree oxygen-sensitive.^6^ Achieving sustained photosynthetic H_2_ production will require both the diversion of a higher proportion of photosynthetic electrons to the hydrogenase and a solution to the problem of hydrogenase inhibition by oxidizing conditions.^7^ Both of these issues pertain to access to electron donors and acceptor efficacy since, although the cyanobacterium *Synechocystis* sp. PCC 6803 (hereafter *Synechocystis*) hydrogenase is O_2_-sensitive,^3^ inactivation can be reversed within minutes if the environment becomes anoxic^4,8^ and significant activity can be maintained in the presence of oxygen if the environment is sufficiently reducing.^5^

We set out to investigate hydrogenase location and activity in the model unicellular cyanobacterium *Synechocystis*; location potentially controlling access of the hydrogenase to the photosynthetic electron transport chain. The bidirectional [NiFe] hydrogenase of *Synechocystis* is a heteropentameric enzyme in which hydrogenase activity is located to the HoxYH subunits, while the HoxEFU subunits constitute a diaphorase for electron transfer to and from the hydrogenase subunits.^8,9^ *In vitro* studies demonstrated that it is truly bidirectional with a slight bias towards H_2_ production rather than consumption.^5^ The physiological role of the hydrogenase is thus bipartite, acting both as a “valve” for the release of excess electrons under anoxic conditions when O_2_ is not available as an electron acceptor^3^ and generating electrons through consumption of H_2_. Under anoxic conditions, H_2_ production serves to dissipate excess electrons derived from either fermentative metabolism in the dark^10^ *via* NifJ/PFOR (pyruvate:flavodoxin/ferredoxin oxidoreductase) and ferrodoxin (Fd),^11^ or the photosynthetic electron transport chain in the light,^3,4,10^ with electrons passing from Photosystem I (PSI) to the hydrogenase *via* Fd.^11^ This revises previous suggestions that electrons were passed from Fd to NADPH *via* Ferredoxin/NADP^+^ reductase (FNR), which was then oxidized by the hydrogenase.^3,4,10^ The sub-cellular localization of the hydrogenase remains contentious. Biochemical analysis indicates a weak association with the thylakoid membranes.^3^ The hydrogenase lacks a membrane spanning region, leading to the suggestion that it interacts with an integral thylakoid membrane complex.^12,13^ This interaction may be *via* the HoxE subunit, which has been postulated to play a role in transferring electrons between the electron transport chain and the hydrogenase.^12^ It has been suggested that the membrane interaction partner is respiratory complex I (NDH-1) based on sequence homology between the HoxEFU subunits and “missing” subunits of cyanobacterial complex I.^14–16^ However, direct evidence for such an interaction is lacking, and there is an alternative explanation for the homology based on the evolutionary origins of complex I.^17^ Recently it has been shown that NDH-1 acts as a ferredoxin:PQ oxidoreductase;^18^ however it is possible that NDH-1 might have multiple input modules one of which could be Fd *via* NdhS and one the hydrogenase.

## Results and discussion

To determine the localization of the bidirectional hydrogenase in *Synechocystis* cells *in vivo*, we created a gene fusion coding for HoxF with Green Fluorescent Protein (GFP) at the C-terminus. This gene fusion was expressed from the native chromosomal *hoxF* locus of *Synechocystis* (ESI Fig. 1†). PCR and Western blots confirmed that the transformant was fully segregated and all HoxF in the mutant was linked to GFP (ESI Fig. 2 and 3†). The majority of HoxF-GFP (70%) was incorporated into fully-assembled hydrogenase complexes, as shown by 2-dimensional PAGE analysis with immunoblotting (ESI Fig. 4†). Given that all Hox subunits (with the exception of HoxE) were detectable only in the soluble fraction (ESI Fig. 5†) we used this fraction for the analysis of complex assembly. H_2_ production in *hoxF-gfp* was comparable to wild-type (WT), both with and without an artificial exogenous electron donor, indicating that hydrogenase activity and physiological electron supply were not perturbed by the GFP tag (ESI Table 1†).

Confocal fluorescence microscopy was used to determine the localization of HoxF-GFP relative to the intracytoplasmic thylakoid membranes as identified from chlorophyll fluorescence.^19,20^ We acquired multiple images per condition and quantified fluorescence within each cell (ESI Fig. 6† and Methods); by restricting to approximately spherical cells (thus excluding cells in the process of division) we estimated the radial fluorescence profile for each cell. This allowed us to distinguish thylakoid association, cytosol localization and cell membrane/periplasm association. Averaging over cells was performed by a radial rescaling of all cells to the 1/2 maximum radius of the chlorophyll intensity. Further, we identified puncta (protein aggregates) on the basis of local differential fluorescence (see Experimental).

**Fig. 1:**
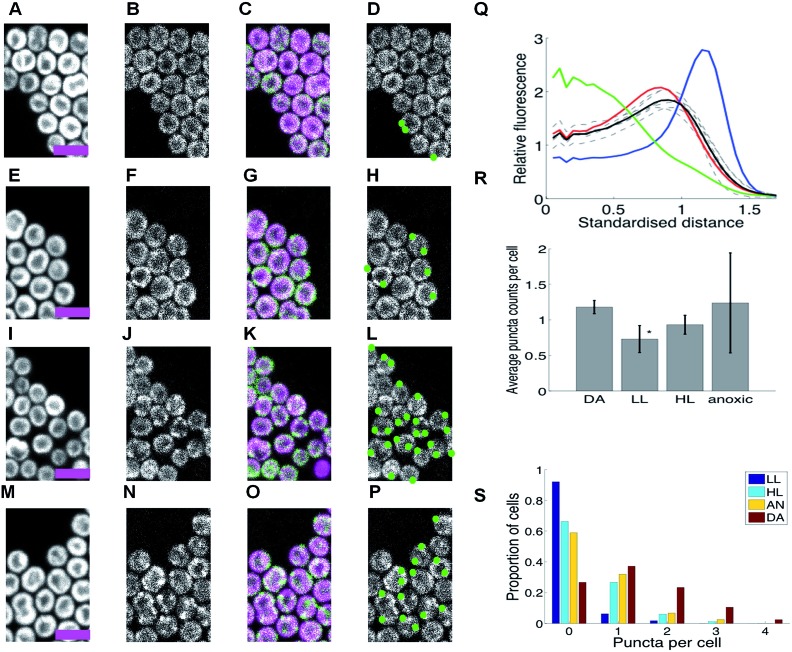
HoxF-GFP distribution and patterning in *Synechocystis* cells grown in low light, high light, under dark adaptation and anoxia (no oxygen). (A–P) Confocal fluorescence micrographs showing chlorophyll fluorescence (first column); GFP fluorescence (second column); chlorophyll/GFP (magenta/green) overlay (third column); puncta of GFP fluorescence (fourth column). Scale-bars 5 microns. (A–D) Cells under low light (LL). (E–H) Cells after 10 min. high-light exposure (HL). (I–L) Cells after dark adaptation (DA) for at least 5 days. (M–P) Cells in anoxic conditions. (Q) Radial distributions of fluorescence for free GFP (green); periplasmic FutA-GFP (blue); chlorophyll (red) and HoxF-GFP after DA (black, mean of 6 replicate experiments individually shown, dashed). Standardised distance refers to rescaling the 1/2 maximum radiuses for chlorophyll to a radial distance of 1. (R) Average counts of puncta/per cell in DA, LL, HL and anoxia with SEM from replicate experiments (6, 7, 6, 2 replicates respectively). * indicates significantly less than DA at *p* < 0.05 (1-tailed MW test). (S) Histograms of puncta counts per cell in a representative experiment from the same batch culture (210 DA, 354 LL, 169 HL, 241 DA cells). Data in (Q-R) from 635-2232 cells by condition. See ESI Fig. 5† for full images.

**Fig. 2:**
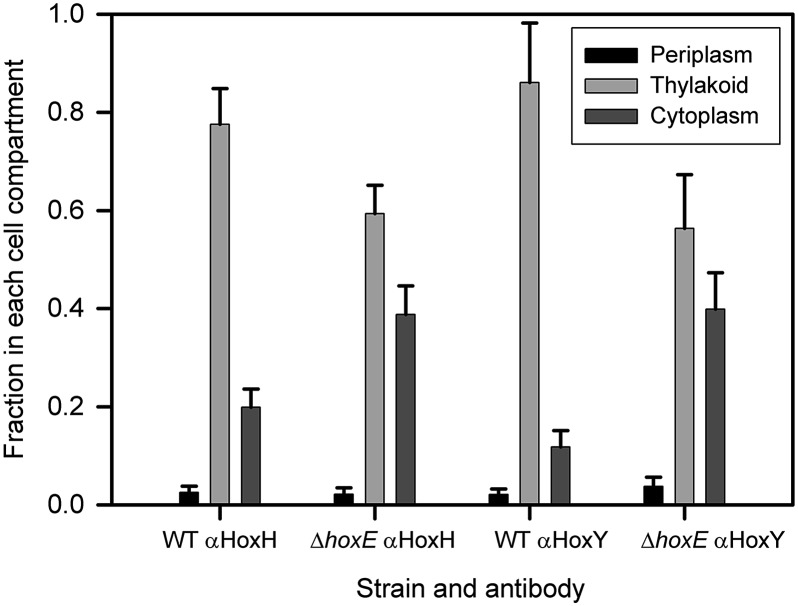
Immunogold electron microscopic localisation of HoxH and HoxY. The histogram shows the fraction of total counts in each cell compartment (periplasm, thylakoid region and cytoplasm) for *Synechocystis* wild-type (WT) and Δ*hoxE* for each primary antibody (cells grown in low light; ± Standard Error). The number of cells counted was 249 for WT, αHoxH; 203 for WT, αHoxY; 81 for Δ*hoxE*, αHoxH; 87 for Δ*hoxE*, αHoxY. Counts have been corrected by subtracting the non-specific background labelling seen in each cell compartment in the Δ*hoxYH* mutant.^12^ See ESI Fig. 8† for images and raw data. Student's *t*-tests indicate that the increased proportion of label in the cytoplasm in Δ*hoxE* is significant (*p* = 0.008 for αHoxH; *p* = 0.00009 for αHoxY).

Under standard low-light (LL, 8 μE m^–2^ s^–1^) aerobic growth conditions, the hydrogenase was localized to the thylakoid membrane (Fig. 1, ESI Fig. 7A†). The radial profile was similar to that of chlorophyll fluorescence; essentially the hydrogenase is distributed within the thylakoid membrane system (Fig. 1A–D and Q). Given that ∼70% of HoxF is assembled into complexes with HoxH and HoxY (ESI Fig. 4†), we would expect these subunits to show a similar distribution to HoxF. Immunogold electron microscopy confirms that the vast majority (∼80%) of HoxH and HoxY are thylakoid membrane-associated in LL wild-type cells (Fig. 2, ESI Fig. 8†), supporting the claim that the hydrogenase complex is likely intact *in vivo*.^11,12^

Incubation of cells under a number of other conditions induced a significant redistribution, with distinct puncta of HoxF-GFP fluorescence forming and locating predominantly at the distal edge of the thylakoid system (Fig. 1). Conditions that induce this redistribution include incubation in high-light (HL, 600 μE m^–2^ s^–1^) for 10 min. (Fig. 1E–H, ESI Fig. 7B†), prolonged incubation (5 days) in the dark (DA) (Fig. 1I–L, ESI Fig. 7C and D†), and within 10 min of anoxia induced by the addition of glucose/glucose oxidase/catalase^21^ (Fig. 1M–P, ESI Fig. 7E†). Puncta counts were highest for the latter two conditions (Fig. 1R and S); of course counts only reflect those visible under our imaging conditions and so our counts are discounted by the visible cellular volume (around 40%). We estimate that individual puncta contain about 5% of the total cell hydrogenase, while on average a total of about 7% of the cellular hydrogenase is in puncta after DA and 10% under anoxia.

Significant photosynthetic H_2_ production has been observed in *Synechocystis* under two related conditions, specifically upon illumination following prolonged DA^4^ and under anoxia induced by glucose/glucose oxidase/catalase,^21^ our conditions in Fig. 1I–L and M–P respectively. Therefore puncta formation of HoxF-GFP is highest under conditions when the hydrogenase is physiologically active for H_2_ production. We considered the possibility that these puncta are localized centers for either biogenesis or catalytic turnover of the hydrogenase complexes. However, it is unlikely that degradation of the hydrogenase would be induced by anoxia, the condition under which activity of the enzyme is best maintained.^4^ Degradation of the hydrogenase also does not seem to be rapid, since there is no discernible loss of HoxF-GFP fluorescence over 90 min following addition of lincomycin (which blocks new protein synthesis),^22^, (ESI Fig. 9†). The rapid effect of anoxia (Fig. 1) suggests that oxygen concentration is one of the controlling factors, potentially through a direct oxygen sensor analogous to those characterized in other bacteria such as *E. coli*,^23^ or more indirectly *via* an effect on the redox state of components of the photosynthetic or respiratory electron transport chains. Redistribution of respiratory complexes in cyanobacterial thylakoid membranes is triggered by changes in the redox state of plastoquinone.^24^ By contrast, the redistribution of the hydrogenase is not influenced by DCMU (3-(3,4-dichlorophenyl)-1,1-dimethylurea) or DBMIB (dibromothymoquinone), (ESI Fig. 10†), which block reduction and oxidation of the PQ pool, respectively, indicating that control of hydrogenase localization is probably *via* a direct redox-sensing mechanism rather than in response to the redox state of electron carriers.

To further explore factors involved in localizing the hydrogenase, we examined the distribution of HoxF-GFP fluorescence in four mutant backgrounds: the Δ*hoxE* and Δ*hoxYH* ^12^ mutants lacking specific hydrogenase subunits, the *hoxE* deletion mutant complemented by *hoxE* overexpression^12^ (denoted *oxhoxE* hereafter), and the M55 mutant lacking respiratory Complex I.^25^ In each case, the *hoxF-gfp* construct was introduced into the appropriate mutant background under the native promoter. PCR and Western blots confirmed that all detectable HoxF in the cells is linked to GFP (ESI Fig. 2 and 11†).

**Fig. 3:**
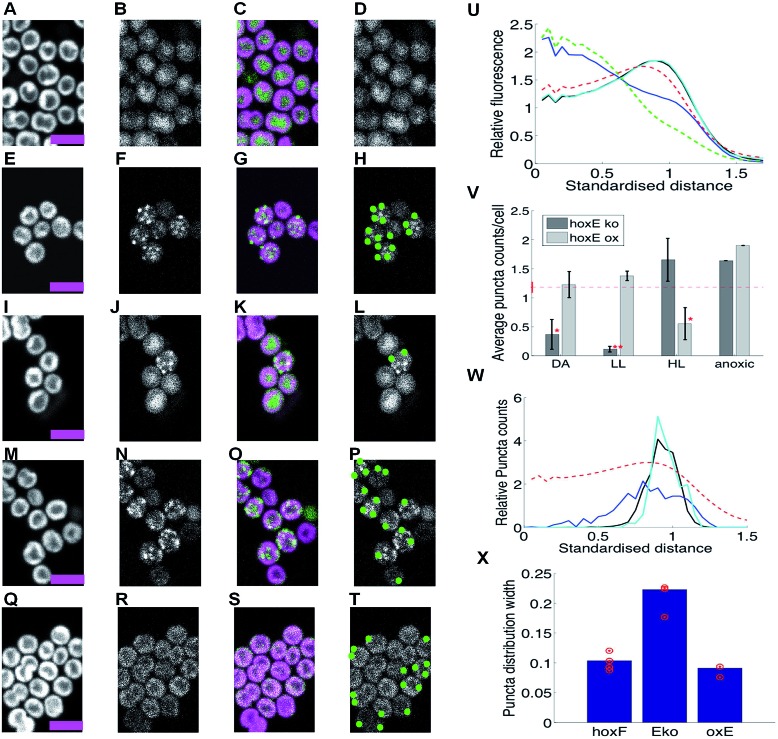
HoxF-GFP distribution and patterning in Δ*hoxE* mutants grown in low light, high light, under dark adaptation and anoxia (no oxygen). (A–D) Δ*hoxE* mutant under low-light (LL). (E–H) Δ*hoxE* mutant after 10 min high light (HL) exposure. (I–L) Δ*hoxE* mutant after 5 days dark adaptation (DA). (M–P) Δ*hoxE* mutant in anoxic conditions. (Q–T) *oxhoxE* mutant in low light (LL). Columns in (A–T) as Fig. 1. Scale-bars 5 microns. (U) Radial distributions of fluorescence under DA conditions for chlorophyll (dashed red); free GFP (dashed green); HoxF-GFP (black, hidden by cyan); HoxF-GFP in Δ*hoxE* (blue); HoxF-GFP in *oxhoxE* (cyan). Standardised distance refers to rescaling the 1/2 maximum radius for chlorophyll to a radial distance of 1. (V) Average counts of HoxF-GFP puncta/cell (mean, SEM) over 2,4,3,1,3,4,2,1 replicate experiments under DA conditions for Δ*hoxE* background (hoxEko) and *oxhoxE* background (Eox). * indicates significantly less than DA HoxF-gfp cells (repeated from Fig. 1R, in red) at *p* < 0.05, while ** indicates significance at *p* < 0.005 (1-tailed MW test). (W) Relative density of HoxF-GFP puncta with respect to radial distance under HL for HoxF-gfp (black, 2206 puncta); Δ*hoxE* background (blue, 635 puncta); *oxhoxE* background (cyan, 203 spots). Radial distribution of chlorophyll fluorescence is shown for reference (dashed red line). Standardised distance refers to rescaling the 1/2 maximum radius for chlorophyll to a radial distance of 1. Data were pooled from replicate experiments. (X) Standard deviation of puncta radial distribution under HL for HoxF-gfp (F), Δ*hoxE* (Eko), *oxhoxE* (oxE++), average over shown (red) replicates (*p* < 10^–8^ for *hoxF-gfp vs.* Δ*hoxE*; *p* = 0.009 for *hoxF-gfp vs. oxhoxE*, F test). Data in U, V from 72–841 cells by condition. See ESI Fig. 9 and 10† for full images and other conditions.

Loss of the HoxE subunit does not impair incorporation of HoxF into HoxFUYH and HoxFU subcomplexes,^12^ nor does it impair hydrogenase activity in the presence of exogenous artificial electron donors.^12,21^ Nevertheless, there are striking changes in the distribution of HoxF-GFP fluorescence in the Δ*hoxE* background (Fig. 3).

In aerobic LL conditions, HoxF-GFP appears dispersed in the cytoplasm, with a radial profile similar to that of free GFP (Fig. 3A–D and U, ESI Fig. 12A†) and very few puncta. This is in sharp contrast to the thylakoid localization of HoxF-GFP in the WT background (Fig. 1). Therefore the HoxE subunit is required for thylakoid membrane-association of the hydrogenase under these conditions. Localisation of HoxH and HoxY by immunogold electron microscopy shows that these subunits also show strong redistribution from the thylakoids to the cytoplasm in the Δ*hoxE* mutant (Fig. 2), confirming that the redistribution of HoxF-GFP fluorescence in Δ*hoxE* reflects relocalisation of the entire hydrogenase complex.

Despite the loss of thylakoid membrane association in Δ*hoxE*, redistribution of the hydrogenase into puncta under HL, prolonged DA and anoxia still occurs in the Δ*hoxE* background (Fig. 3, ESI Fig. 12B–E†), as observed in the WT background (Fig. 1). However, there are differences in redistribution dynamics and the strength of puncta formation; under DA, puncta formation is slower in Δ*hoxE* than in the WT background (ESI Fig. 12C–D† showing that puncta formation occurred between days 3 and 5, whilst puncta formed by day 3 in HoxF-GFP ESI Fig. 7C†), while puncta counts are around 20% higher in HL and anaerobic conditions than in DA, (Fig. 3V). Puncta confinement to the distal thylakoid is also weaker than in the HoxF-GFP strain, (Fig. 3W and X). These differences suggest that although recruitment of the hydrogenase to puncta can occur from the cytosol, recruitment from the thylakoid membrane fraction is faster and gives rise to a tighter localisation.

To confirm that the changes in hydrogenase distribution in Δ*hoxE* are indeed due to loss of the HoxE subunit, we examined the distribution of the hydrogenase in the *oxhoxE* background, in which overexpressed *hoxE* complements the null mutation.^12^ Overexpression of *hoxE* restored the predominant thylakoid localization of HoxF-GFP fluorescence (Fig. 3Q–T for LL, ESI Fig. 13A–D†), and the localisation of HoxF-GFP puncta to the distal thylakoid (Fig. 3W and X). Puncta were formed under LL, HL, prolonged DA and anoxia (Fig. 3V). The only clear difference from the WT background was the greater number of puncta detected in LL adapted cells and fewer in HL in the *oxhoxE* background (compare Fig. 1R and 3V), presumably a result of higher *hox* operon and HoxE expression.^12^ This suggests that over-expression of HoxE has shifted the phenotype under light exposure, higher light levels being required to reduce puncta counts; in effect high HoxE inhibits the effect of low light signals in relocalizing the hydrogenase to the thylakoid membranes.

**Fig. 4:**
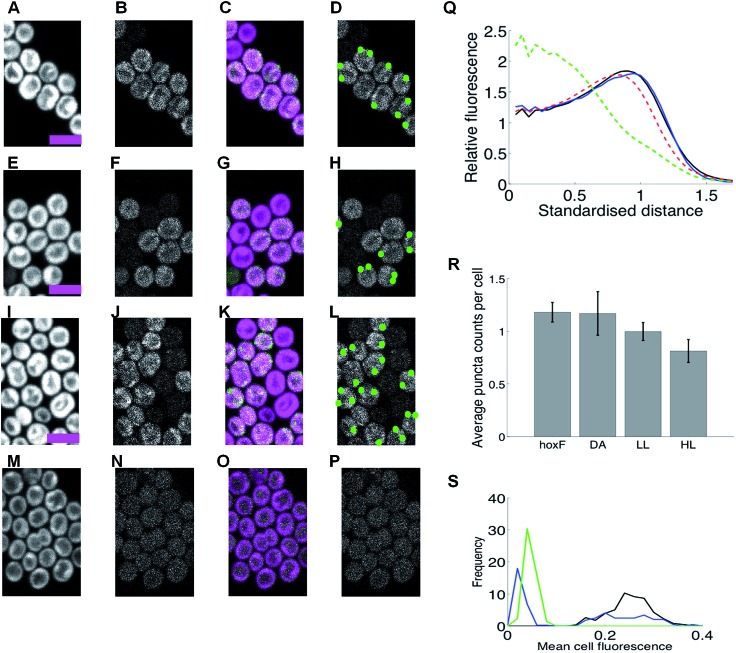
HoxF-GFP distribution and patterning in M55 (Complex I-deficient) and Δ*hoxHY* mutants grown in low light, high light, under dark adaptation and anoxia (no oxygen). (A–D) M55 under low light (LL). (E–H) M55 after 10 min high light exposure (HL). (I–L) M55 after 5 days dark adaptation (DA). (M–P) Δ*hoxYH* under low light (LL). Columns in (A–P) as Fig. 1. Scale-bars 5 microns. (Q) Radial distribution of fluorescence: free GFP (green); chlorophyll (red) HoxF-GFP (black); HoxF-GFP in M55 background (expressing cells, blue). Standardised distance refers to rescaling the 1/2 maximum radius for chlorophyll to a radial distance of 1. (R) Mean counts of HoxF-GFP puncta/cell in M55 background under DA, HL, LL and anoxia with SEM over replicate experiments (6, 2, 2, and 2 respectively). DA HoxF-gfp cells repeated from Fig. 1R, (S) Mean HoxF-GFP fluorescence per cell under LL for HoxF-GFP (black), M55 background (blue) and Δ*hoxYH* background (green). Fluorescence is corrected for cell autofluorescence by subtracting cell fluorescence after photobleaching. Data in Q–R from 291–492 cells by condition.

Eckert *et al.* ^12^ demonstrated that levels of HoxE, HoxF, and HoxU are decreased by up to 25–70% of WT in HoxYH subcomplex mutants. In the Δ*hoxYH* background, HoxF-GFP fluorescence per cell is extremely low, being decreased by a factor of about 5 in LL (Fig. 4M–P and S, ESI Fig. 14A†) and similarly in other conditions (ESI Fig. 14B and C†). Δ*hoxYH* lost the HoxF-GFP thylakoid association characteristic of the WT, with only occasional puncta (ESI Fig. 14†). Expression was too low for accurate quantification or condition dependencies. This mutant does not form a functional hydrogenase, but the diaphorase subunits can form a HoxEFU sub-complex.^12^ However, the low HoxF fluorescence in the Δ*hoxYH* background suggests that any subcomplexes formed are unstable, fail to localize to the thylakoid, and are rapidly turned over *in vivo*.

In the complex I-deficient M55 background about 50% of cells showed no HoxF-GFP fluorescence (Fig. 4), suggesting a stochastic disruption of *hox* gene expression and/or Hox protein stability. However the cells with no HoxF-GFP are still alive: this is evident from their strong chlorophyll signal (Fig. 4). This is consistent with Gutthann *et al.* ^10^ who noted there was poor hydrogenase activity in the M55 mutant compared to the WT. However, in those cells in which HoxF-GFP was evident, levels (Fig. 4S†) and localization were generally similar to those seen in the WT background, both with regard to thylakoid membrane association and formation of puncta (Fig. 4A–L and Q–R), although redistribution to puncta appeared disrupted under light to some degree.

Our results show that there are at least two localization signals for the hydrogenase in *Synechocystis* – a HoxE-dependent interaction which anchors the majority of the hydrogenase to the thylakoid membrane, and a second aggregation signal (triggered under specific conditions such as anoxia) that partially localizes the hydrogenase to discrete puncta (Fig. 1). These puncta are strongly targeted to the distal thylakoids (Fig. 1H, L and P, 3W). Our data suggests that both populations are functional given that the thylakoid dispersed population is in the majority, whilst puncta formation requires a specialized aggregation mechanism. The fact that puncta formation is highest under conditions when hydrogen is produced (DA, anoxia) implies that the puncta are related to hydrogen production. The formation of puncta is independent of the HoxE subunit (Fig. 3), but this subunit influences the distribution of puncta within the thylakoid membrane, since targeting to the distal edge of the thylakoid system is weaker in Δ*hoxE* (Fig. 3), and over-expression appeared to inhibit light dependent localization signals, (Fig. 1R and 3V). The aggregation mechanism is unclear. Since the HoxE subunit, that anchors the hydrogenase to the thylakoid membrane, is not essential for puncta formation it seems unlikely that membrane curvature is the driving force, a mechanism suggested for row formation of the F_1_F_0_-ATP synthase in mitochondrial cristae.^26^ In addition, we showed that loss of NDH-1 has no effect on the thylakoid localization of Hox. The putative docking of hydrogenase with NDH1 would require the NdhHIJK subunits linked to NdhB.^27^ These subunits are absent in the M55 mutant and their loss has no effect on the thylakoid localization of Hox under normal LL conditions (Fig. 3). Therefore NDH-1 cannot be the main thylakoid interaction partner of HoxE.

The majority of HoxF-GFP (∼70%) is incorporated into fully-assembled HoxEFUYH/HoxFUYH complexes with a small amount (∼23%) forming sub complexes (HoxEFU and HoxFU) similar to Eckert *et al.* ^12^ However, it is not absolutely clear whether these sub-complexes exist *in vivo* as they have only been observed *in vitro* following isolation of the complex. In fact, pentameric HoxEFUYH hydrogenases tend to dissociate following cell breakage into the diaphorase (Hox-EFU) and hydrogenase (HoxYH) subunits.^28,29^ Gutekunst *et al.* ^11^ have demonstrated that the pentameric complex is extremely fragile and easily breaks upon cell rupture in *Synechocystis*. Therefore it may be impossible to isolate the enzyme in its native form. The correlation we observed between spatial location of HoxF-GFP fluorescence and HoxH and HoxY as judged by immunogold electron microscopy (Fig. 2), particularly under Δ*hoxE* (Fig. 2 and 3) strongly suggests that the hydrogenase complex is intact *in vivo*, and that HoxF-GFP fluorescence is a good indicator of hydrogenase location.

Physiologically regulated hydrogenase relocalization adds a new dimension to hydrogenase functionality-redistribution between the thylakoid dispersed and puncta populations could potentially optimise access to electron donors in a condition dependent manner. The strong HoxE-dependent thylakoid association suggests that access to a membrane localized electron donor is crucial, either during fermentation or whilst coupling to the photosystem electron pathway. Of fundamental relevance is the presence of soluble pools of reductant, specifically NADPH (from the photosystems) and NADH (from fermentation), suggesting that *in vivo* the hydrogenase is inefficient at coupling to these pools. Utilization of membrane localized donors is thus indicated; this is consistent with the *in vitro* results of Gutekunst *et al.* who demonstrate that flavodoxin and ferredoxin (Fd) are the main electron donors to the hydrogenase.^11^ Our data suggests that the reduced form of these donors is localized near the thylakoids under hydrogen producing conditions. Thylakoid membrane localization would obviously place the hydrogenase in closer proximity to the supply of reductant generated from the photosynthetic electron transport chain, specifically reduced Fd thereby providing a rapid oxidation of this local pool and hence a release of excess electrons. Evidence that specific localization is important also comes from the increased formation of puncta under conditions when physiological H_2_ photoproduction can be observed (DA and anoxia). The localization of these puncta to a specific site in the distal thylakoid is particularly interesting, distant from the carboxysomes in the central cytosol^30^ but appearing to retain attachment to the thylakoids. The puncta could thus represent two functional populations: (1) a hydrogenase population localized to patches close to photosynthetic electron transport, thereby enhancing the efficacy of electron dispersal through some form of substrate channeling, or (2) a population optimized for hydrogen uptake following high light exposure as the puncta are further away from the source of reduced Fd compared to the thylakoid-associated population, thereby favoring the back-reaction. Either of these could explain the evolution of mechanisms to dynamically separate the hydrogenase into two populations.

## Conclusions

Our results indicate that mechanisms for rapid physiological control of hydrogenase location in response to oxygen level and electron transport activity (*e.g*. HL) are present and demonstrate that the sub-cellular localization of the hydrogenase is important for physiological electron supply. Taken together, this raises the possibility that termination of H_2_ production *in vivo* is not solely due to inactivation by oxygen: it could also be a consequence of physiological regulation of hydrogenase location and electron supply in response to redox levels. This would make physiological sense, as the hydrogenase is presumably only required as an emergency electron sink in the absence of oxygen when terminal oxidases cannot function.^10^ Circumventing this emergency role and instituting continuous production is the major issue in photosynthetic hydrogen production. Determination of the molecular basis for control of hydrogenase location could thus pave the way to engineering improved cyanobacterial cells for solar-powered bio-hydrogen production.

## Experimental

### Bacterial strains and media

Wild-type *Synechocystis* sp. PCC 6803 (glucose tolerant strain), M55, Δ*hoxYH*, Δ*hoxE* and the Δ*hoxE* over expressed strain (*oxhoxE*)^12,25^ were grown photoautrophically in BG-11 medium^31^ at 30 °C. For our standard low-light (LL) conditions cells were grown under 8 μmol m^–2^ s^–1^ white light in tissue culture flasks (Nunc UK), with continuous shaking. For dark adapted conditions (DA) cells were grown at 30 °C in tissue culture flasks wrapped in foil for 5 days, before being spotted in low light onto BG-11 plates. High light (HL) cells were spotted onto BG-11 plates and illuminated for 10 min under 600 μmol m^–2^ s^–1^ white light. For anoxic conditions, catalase (500 U), glucose (5 mM), and glucose oxidase (30 U) were added to LL cells to make the medium totally anaerobic before being spotted onto BG-11 agar plates. *E*.*coli* strains used in this study were DH5α and BW25113 (*E. coli* stock centre). LL cultures were incubated for 1 h with 20 μM DCMU and 5 μM DBMIB. Lincomycin was added at 100 μg ml^–1^ to DA cells prior to HL exposure.

### Transformation of *Synechocystis* sp. PCC 6803

*Synechocystis* sp. PCC 6803 cells were transformed according to.^32^ Briefly a culture in exponential growth was harvested and washed with fresh BG-11 and re-suspended to give a final concentration of 1 × 10^9^ cells per ml. Approximately 10 μl of plasmid DNA was then added to 150 μl of cells and incubated at 50 μmol m^–2^ s^–1^ white light at 30 °C for 1–5 hours before being spread onto BG-11 plates. The plates were then incubated under 50 μmol m^–2^ s^–1^ white light at 30 °C until confluent green growth was observed (approximately 16 hours). Increasing amounts of apramycin were then added; cells were further grown on selective plates containing a final concentration of 50 μg ml^–1^ apramycin.

### Generation of a GFP tagged HoxF protein in *Synechocystis* sp. PCC 6803

The HoxF-GFP strain was generated as detailed in the REDIRECT manual^33^ with minor modifications. The protocol, plasmids and strains were provided by PBL Biomedical Laboratories. The forward *hox*F (5′acgactcaagtccacatagg3′) and the reverse *hoxF* (5′caccagggtggaagctaaac3′) primers were used to amplify a 3 kb region, which included the *hoxF* gene flanked by 1 kb either side, to assist with homologous recombination. The 3 kb PCR products were then cloned into the pGEM T-easy vector (Promega) as detailed in the Promega manual. HoxF-GFP fusions were generated by amplifying the apramycin GFP cassette from the plasmid pIJ786 using two long PCR primers

*hoxFRF*-5′agttgattttttgatttgttgttattgagcttaaacccc**ctgccgggcccggagctgcc**3′

*hoxFRR*-5′gtgtttcagaaaagttaactgagtggataaattaccgaa**attccggggatccgtcgacc**3′

Each individual primer has at the 5′ end 39nt matching *Synechocystis* sequence either side of but not including the stop codon and a 3′ sequence (19nt or 20nt) matching the right or left end of the cassette. A full in frame GFP fusion was generated *via* homologous recombination leading to the incorporation of the GFP and a 21nt linker region (**ctgccgggcccggagctgccg**) at the C-terminus of the HoxF protein. Successful transformants were screened *via* PCR using the primers (*hoxFFS*-5′tatgaagaattactcaaagtc 3′ and *hoxFRS* 5′ acaatacctgttccagagggg 3′) and sequenced using T7 and S6 primers (Promega).

### Protein analysis and immunoblotting

The soluble fraction from each strain was isolated by glass bead breakage and differential centrifugation. For ESI Fig. 5† both soluble and cell pellet fractions were prepared according to Eckert *et al.* ^12^ For each strain, a 50 ml liquid culture was grown to stationary phase and the cells were harvested, re-suspended and washed twice in ACA buffer (750 mM ε-amino caproic acid, 50 mM BisTris/HCL pH = 7.0, 0.5 mM EDTA). The final volume of cell suspension was ∼500 μl and ∼200 μl of glass beads (212 to 300 μm in diameter, Sigma-Aldrich, UK) were added. Cells were broken with a custom made vortexer at 4 °C with a 5 min on/5 min off cycle. After cell breakage the sample was centrifuged for 10 min at maximum speed in a microfuge and then again in an ultracentrifuge at 100 000 × *g* for 30 min at 4 °C. The resulting supernatant, *i.e.* the soluble fraction, was normalized according to its OD_650_ (typically, the OD_650_ of a 1 : 10 dilution was measured and a dilution factor calculated on the basis to obtain an OD_650_ of 0.02 for the 1 : 10 dilution). To solubilise any remaining thylakoid membrane fragments, *n*-dodecyl-d-maltoside (*n*-DM) was added to a final concentration of 0.5% (w/v) from a 10% (w/v) stock and as a last step the same volume of Coomassie loading solution (750 mM ε-amino caproic acid, 5% (w/v) Coomassie-G) was added. 22 μl of sample were loaded per lane on a 8 to 12 % (w/v) linear polyacrylamide (PAA) gradient Blue-native (BN) PAGE first dimension gel and the electrophoretic run was performed according to.^34^ 2D SDS PAGE gels were run using 17.5% (w/v) PAA and 6 M urea containing gels as described in Boehm *et al.* ^34^ Gels were either Coomassie-stained, silver-stained^35^ or electro-blotted onto nitrocellulose membrane using the iBlot system (Invitrogen, UK) according to the manufacturer's instructions. Immunoblotting analyses were performed using specific primary antibodies and a horseradish peroxidase-conjugated secondary antibody (GE Healthcare, UK). Signals were visualised using a chemiluminescent kit (SuperSignal West Pico, Pierce, USA). Primary antibodies used in this study were: (i) antiHoxE (directed against *E. coli* over expressed HoxE), (ii) antiHoxF (directed against *E. coli* over expressed HoxF), (iii) antiHoxH (directed against *E. coli* over expressed HoxH), (iv) antiHoxU (directed against *E. coli* over expressed HoxU), (v) antiHoxY (directed against *E. coli* over expressed HoxY) and (vi) antiGfp (Gentaur Molecular Products, Belgium). Blots were analysed using Image J software to semi quantify individual bands.

### Confocal microscopy

Cells were immobilized by absorption onto blocks of BG-11 agar in a custom built sample holder using a LeicaTCS-SP5 with a 60× oil immersion objective (NA 1.4) with excitation at 488 nm from an argon laser. GFP fluorescence was recorded at 502–512 nm and chlorophyll fluorescence was recorded at 670–720 nm. The confocal pinhole was set to give a z-resolution of about 0.8 μm. Images were recorded over 5 minute periods for each sample.

## Hydrogenase assay

As described in (ref. 12).

### Regional fluorescence quantification

Chlorophyll fluorescence was used to demarcate cell boundaries. Specifically, object extraction was performed using a threshold that maximized the number of objects identified as cells (based on limits for cell area, cell eccentricity and a second (higher) threshold to define the inner cytosol region). Cells that were in the process of division were thus excluded from the analysis (eccentricity threshold) as were dead/dying cells that had low chlorophyll fluorescence. The bleached image was used to calibrate the auto fluorescence, *i.e*. levels of GFP protein were defined as relative to the bleached image. Radial fluorescence were determined on a per cell basis by using the chlorophyll fluorescence to define the cell geometry, *i.e*. radial coordinates were used to allow cell fluorescence to be averaged over rotation angle in each cell. Averaging over cells was performed by rescaling radially relative to the 1/2 maximum radius of the chlorophyll intensity, the standardised distance. Spots were determined by using a local fluorescence filter. Spot counts post-bleach and in wild-type were used to calibrate detection thresholds. Detection thresholds for cells and spots varied only slightly between experiments. All software was written in MatLab.

### Puncta quantification and statistical tests

Puncta were determined by using a local ratio of the fluorescence image filtered with Gaussian filters (standard deviations of 3.5 and 6.5 pixels) and a ratio threshold of 1.65. Puncta counts post-bleach and in wild-type were used to calibrate detection thresholds to minimise false positives. Detection thresholds for cells and puncta varied only slightly between experiments. Puncta counts per cell (defined above) were determined for each image data set (100 s of cells, see figure legends). Statistical significance for puncta counts was determined by Mann–Whitney tests. All software was written in MatLab.

### Immunogold electron microscopy

Cell suspensions were fixed for 15 min at room temperature with 3% (w/v) paraformaldehyde in 100 mM phosphate buffer pH 7.3. To remove the fixative, cells were washed three times with 100 mM phosphate buffer. After embedding in 1% (w/v) low-gelling temperature agarose, samples were cut into 1–2 mm cubic blocks, dehydrated through a graded ethanol series (15 min 30%, 15 min 50%, 15 min 70%, 15 min 90% and 3 × 20 min 100%) and embedded in LR White resin.^36^ Thin sections were cut with a glass knife at a Reichert Ultracut E microtome, collected on nickel grids coated with pioloform, etched for 5 min with H_2_O_2_ (5% w/v) and washed in phosphate-buffered saline (PBS) before blocking with Bovine Serum Albumen (BSA; 10% w/v in PBS) for 1 h at room temperature. Sections were then incubated for 1 h at room temperature with rabbit primary antibodies against HoxH or HoxY,^12^ diluted to 1 : 250 and 1 : 100 respectively in blocking buffer. After washing in PBS (6 × 2 min.) samples were incubated for 1 h with goat anti-rabbit IgG conjugated with 10 nm colloidal gold particles (Sigma-Aldrich) at 1 : 50 dilution in blocking buffer, and then washed with blocking buffer (3 × 2 min.), PBS (3 × 2 min.) and ultrapure water (3 × 1 min.).^37^ Sections were post-stained with saturated aqueous uranyl acetate and air-dried before examination in a JEOL JEM-1230 transmission electron microscope at an accelerating potential of 80 kV.

## References

[cit1] Vignais P. M., Billoud B. (2007). Chem. Rev..

[cit2] Ghirardi M. L., Dubini A., Yu J., Maness P. C. (2009). Chem. Soc. Rev..

[cit3] Appel J., Phunpruch S., Steinmüller K., Schulz R. (2000). Arch. Microbiol..

[cit4] Cournac L., Guedeney G., Peltier G., Vignais P. M. (2004). J. Bacteriol..

[cit5] McIntosh C. L., Germer F., Schulz R., Appel J., Jones A. K. (2011). J. Am. Chem. Soc..

[cit6] Tamagnini P., Axelsson R., Lindberg P., Oxelfelt F., Wünschiers R., Lindblad P. (2002). Microbiol. Mol. Biol. Rev..

[cit7] Abou Hamdan A., Burlat B., Gutiérrez-Sanz O., Liebgott P. P., Baffert C., De Lacey A. L., Rousset M., Guigliarelli B., Léger C., Dementin S. (2013). Nat. Chem. Biol..

[cit8] Germer F., Zebger I., Saggu M., Lendzian F., Schulz R., Appel J. (2009). J. Biol. Chem..

[cit9] Schmitz O., Boison G., Salzmann H., Bothe H., Schütz K., Wang S. H., Happe T. (2002). Biochim. Biophys. Acta.

[cit10] Gutthann F., Egert M., Marques A., Appel J. (2007). Biochim. Biophys. Acta.

[cit11] Gutekunst K., Chen X., Schreiber K., Kaspar U., Makam S., Appel J. (2014). J. Biol. Chem..

[cit12] Eckert C., Boehm M., Carrieri D., Yu J., Dubini A., Nixon P. J., Maness P. C. (2012). J. Biol. Chem..

[cit13] Tamagnini P., Leitão E., Oliveira P., Ferreira D., Pinto F., Harris D. J., Heidorn T., Lindblad P. (2007). FEMS Microbiol. Rev..

[cit14] Appel J., Schulz R. (1996). Biochim. Biophys. Acta.

[cit15] Prommeenate P., Lennon A. M., Markert C., Hippler M., Nixon P. J. (2004). J. Biol. Chem..

[cit16] Schmitz O., Bothe H. (1996). Naturwissenschaften.

[cit17] Marreiros B. C., Batista A. P., Duarte A. M., Pereira M. M. (2013). Biochim. Biophys. Acta.

[cit18] Battchikova N., Wei L., Du L., Bersanini L., Aro E. M., Ma W. (2011). J. Biol. Chem..

[cit19] Mullineaux C. W., Sarcina M. (2002). Trends Plant Sci..

[cit20] Spence E., Sarcina M., Ray N., Møller S. G., Mullineaux C. W., Robinson C. (2003). Mol. Microbiol..

[cit21] Aubert-Jousset E., Cano M., Guedeney G., Richaud P., Cournac L. (2011). FEBS J..

[cit22] Komenda J., Hassan H. A., Diner B. A., Debus R. J., Barber J., Nixon P. J. (2000). Plant Mol. Biol..

[cit23] Tuckerman J. R., Gonzalez G., Sousa E. H., Wan X., Saito J. A., Alam M., Gilles-Gonzalez M. A. (2009). Biochemistry.

[cit24] Liu L.-N., Bryan S. J., Huang F., Yu J., Nixon P. J., Rich P. R., Mullineaux C. W. (2012). Proc. Natl. Acad. Sci. U. S. A..

[cit25] Ogawa T. (1991). Proc. Natl. Acad. Sci. U. S. A..

[cit26] Davies K. M., Anselmi C., Wittig I., Faraldo-Gómez J. D., Kühlbrandt W. (2012). Proc. Natl. Acad. Sci. U. S. A..

[cit27] Zhang P., Battchikova N., Jansen T., Appel J., Ogawa T., Aro E. M. (2004). Plant Cell.

[cit28] Palágyi-Mészáros L. S., Maróti J., Latinovics D., Balogh T., Klement E., Medzihradszky K. F., Rákhely G., Kovács K. L. (2009). FEBS J..

[cit29] Serebriakova L. T., Sheremet'eva M. E. (2006). Biochemistry.

[cit30] So A. K., John-McKay M., Espie G. S. (2002). Planta.

[cit31] Rippka R., Deruelles J., Waterbury J., Herdman M., Stanier R. (1979). J. Gen. Microbiol..

[cit32] Chauvat F., Rouet P., Bottin H., Boussac A. (1989). Mol. Gen. Genet..

[cit33] Gust B., Challis G. L., Fowler K., Kieser T., Chater K. F. (2003). Proc. Natl. Acad. Sci. U. S. A..

[cit34] Boehm M., Nield J., Zhang P., Aro E. M., Komenda J., Nixon P. J. (2009). J. Bacteriol..

[cit35] Blum H., Beier H., Gross H. J. (1987). Electrophoresis.

[cit36] Bowling A. J., Vaughn K. C. (2008). J. Microsc..

[cit37] Klint J., Rasmussen U., Bergman B. (2007). J. Plant Physiol..

