# Supplementary material for: Solar powered biohydrogen production requires specific localization of the hydrogenase
Source: Energy Environ Sci. 2014 Sep 23;7(11):3791–800. doi: 10.1039/c4ee02502d (PMC4535174; doi:10.1039/c4ee02502d)
Supplement: Supplementary file 1 [file EE-007-C4EE02502D-s001.pdf]

Electronic Supplementary Material (ESI) for Energy & Environmental Science.
This journal is © The Royal Society of Chemistry 2014

Supplementary Data

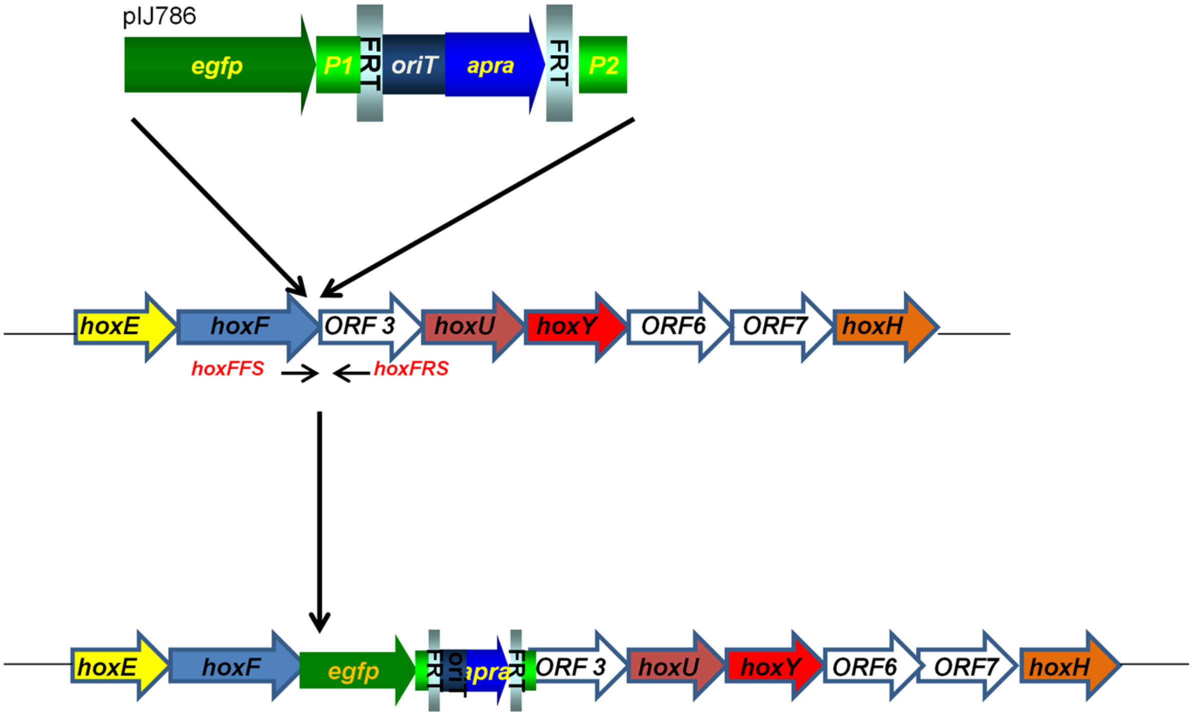

Supplementary Figure 1. Chromosomal map of the *hox* operon in *Synechocystis* sp. PCC 6803. Schematic showing integration of the *eGFP* cassette at the C-terminus of *hoxF*. OriT(RK2) in the disruption cassette stands for origin of transfer and allows for conjugation. The cassette also includes a 19bp and 20bp primer site called P1 and P2 respectively. These are identical in all disruption cassettes. The resistance genes are also flanked by FRT sites (FLP-mediated recognition targets) which allow FLP-mediated excision of the disruption cassette. The size of the cassette is~ 2100bp. The primers *hoxFFS* and *hoxFRS* were used to check for complete segregation of the cassette.

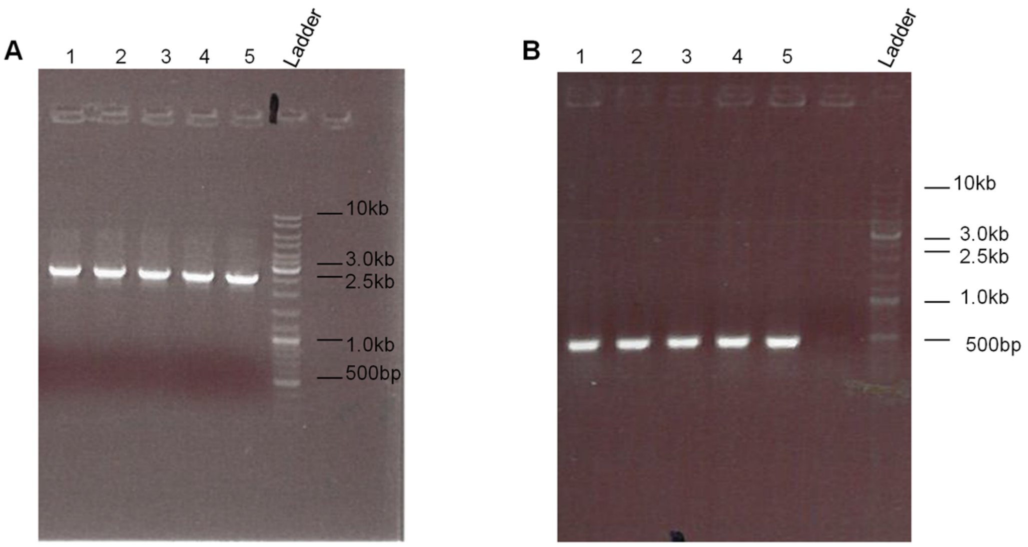

Supplementary Figure 2. Confirmation of complete segregation of *hoxF-GFP* in *Synechocystis* by PCR. A. Agarose gel showing full segregation of Synechocystis *hoxF-GFP*. Primers were designed to amplify between the 3' end of the *hoxF* gene and just downstream of the *hoxF* gene (*orf3)*. The inclusion of the GFP apramycin cassette increases the size of the product to ~2.5kb. Lane 1: *hoxF-GFP*, Lane 2: *hoxF-GFP* in *ΔhoxE* , Lane 3: *hoxF-GFP* in *ΔhoxE* /*hoxE* ox, Lane 4: *hoxF-GFP* in *ΔhoxHY* and Lane 5: *hoxF-GFP* in M55. Lane 6: DNA ladder mix100-10,000bp (PeqLab). **B** Agarose gel showing the WT product which is 403bp in each of the individual strains. Lane 1: WT, Lane 2: *ΔhoxE*, Lane 3: *ΔhoxE* /*hoxE* ox, Lane 4: *ΔhoxHY* and Lane 5: M55. Lane 6: DNA ladder mix 100-10,000bp (PeqLab).

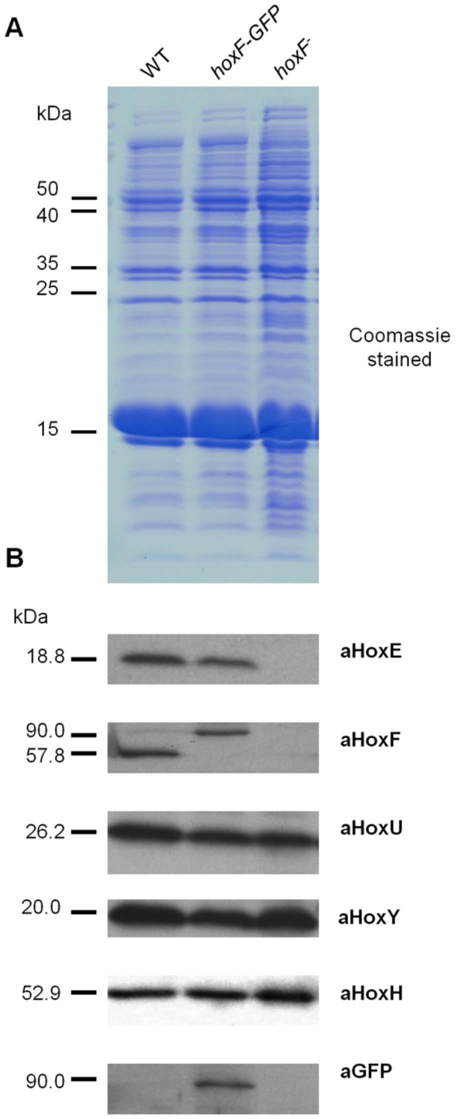

C

Subunit	WT %	*hoxF*-GFP %
HoxE	100	85
HoxF	100	92
HoxU	100	94
HoxY	100	89
HoxH	100	85

Supplementary Figure 3. Confirmation of complete segregation of HoxF-GFP in *Synechocystis* (**A**) SDS PAGE gel of soluble whole cell protein extracts generated from the wild-type, *hoxF-GFP*, and Δ*hoxF* strains. (**B**) Immunoblotting analysis on the same strains using HoxE, F, H, U, Y and GFP antibodies. The presence of a higher molecular weight signal for the HoxF antibody in the *hoxF-GFP* strain and the signal for the GFP antibody confirm the successful GFP tagging of the HoxF subunit. Complete segregation is confirmed by the absence of the signal for HoxF that is present in the WT. (**C**) Semi quantification of the structural subunits in *hoxF-GFP* relative to WT using Image J software. The GFP cassette has had little effect on the expression at the protein level of the structural subunits of the hydrogenase complex with levels of HoxE, F, U, Y and H accumulating to levels similar to those seen in the WT.

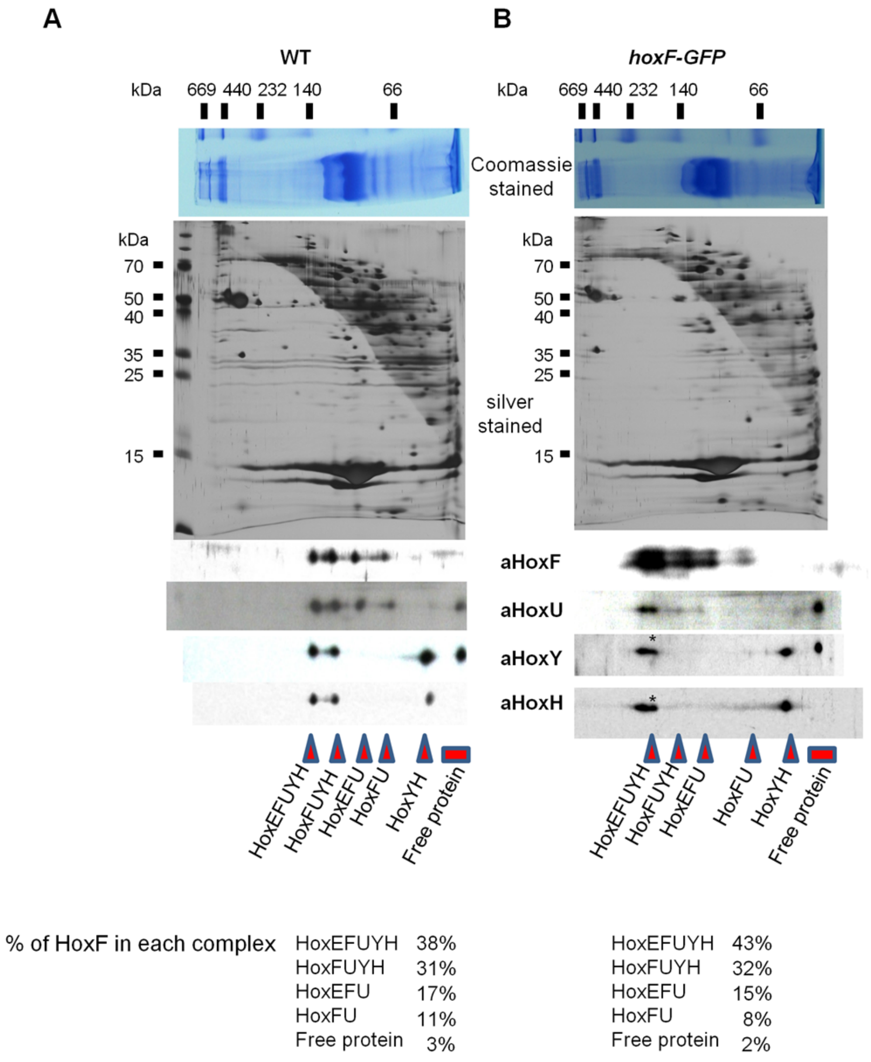

Supplementary Figure 4. GFP-tagged HoxF can assemble into pentameric HoxE(F-GFP)UYH complexes. 2D-BN/SDS PAGE analyses of *Synechocystis* soluble whole cell protein extracts followed by immunoblotting with antibodies specific to the HoxF,U,Y and H subunits. Comparison between the (**A**) wild-type and (**B**) the *hoxF-GFP* strains demonstrates that the majority of HoxF-GFP can be incorporated into fully-assembled hydrogenase complexes (HoxEFUYH and HoxFUYH). Semi quantification of proteins was carried out using Image J software based on band intensity. * Indicates the HoxFUYH complex.

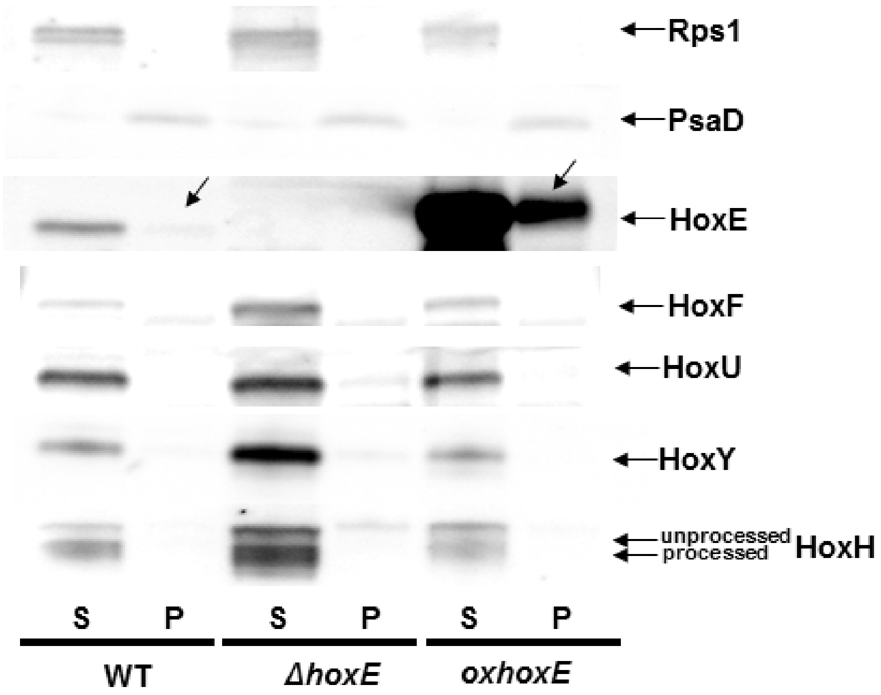

Supplementary Figure 5. Hox subunits are located in the soluble fraction with only HoxE detected in the whole membrane pellet. Immunoblotting analysis of soluble protein extracts (S) and pellet protein extracts (P) generated from wild-type (WT), *ΔhoxE*, and *oxhoxE* strains using HoxE, F, H, U, Y, PsaD and Rps1 antibodies. Only the HoxE subunit can be detected in the pellet (arrows).

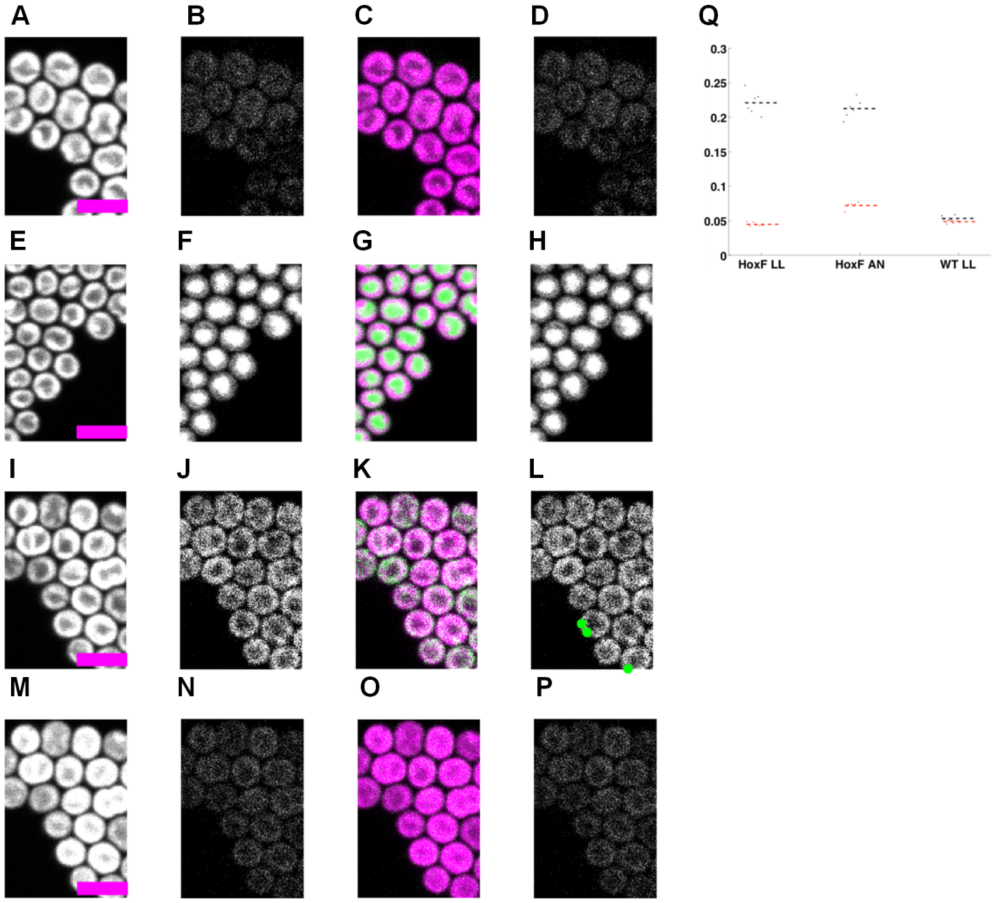

Supplementary Figure 6. Fluorescence quantification for protein location. Confocal fluorescence micrograph strips showing chlorophyll fluorescence (first column), GFP (second), the chlorophyll/GFP (magenta/green) overlay (third) and located puncta in green (fourth). WT cells under LL (**A-D**), free GFP under LL exposure (**E-H**), HoxF-GFP cells under LL, as Fig. 1, (**I-L**) and after bleaching (**M-P**). (**Q**) Fluorescence intensity before (black) and after bleaching (red) for HoxF-GFP cells (under LL, anoxic (AN)) and WT (LL). Six image plates shown for each (dots) and their mean (dashed).

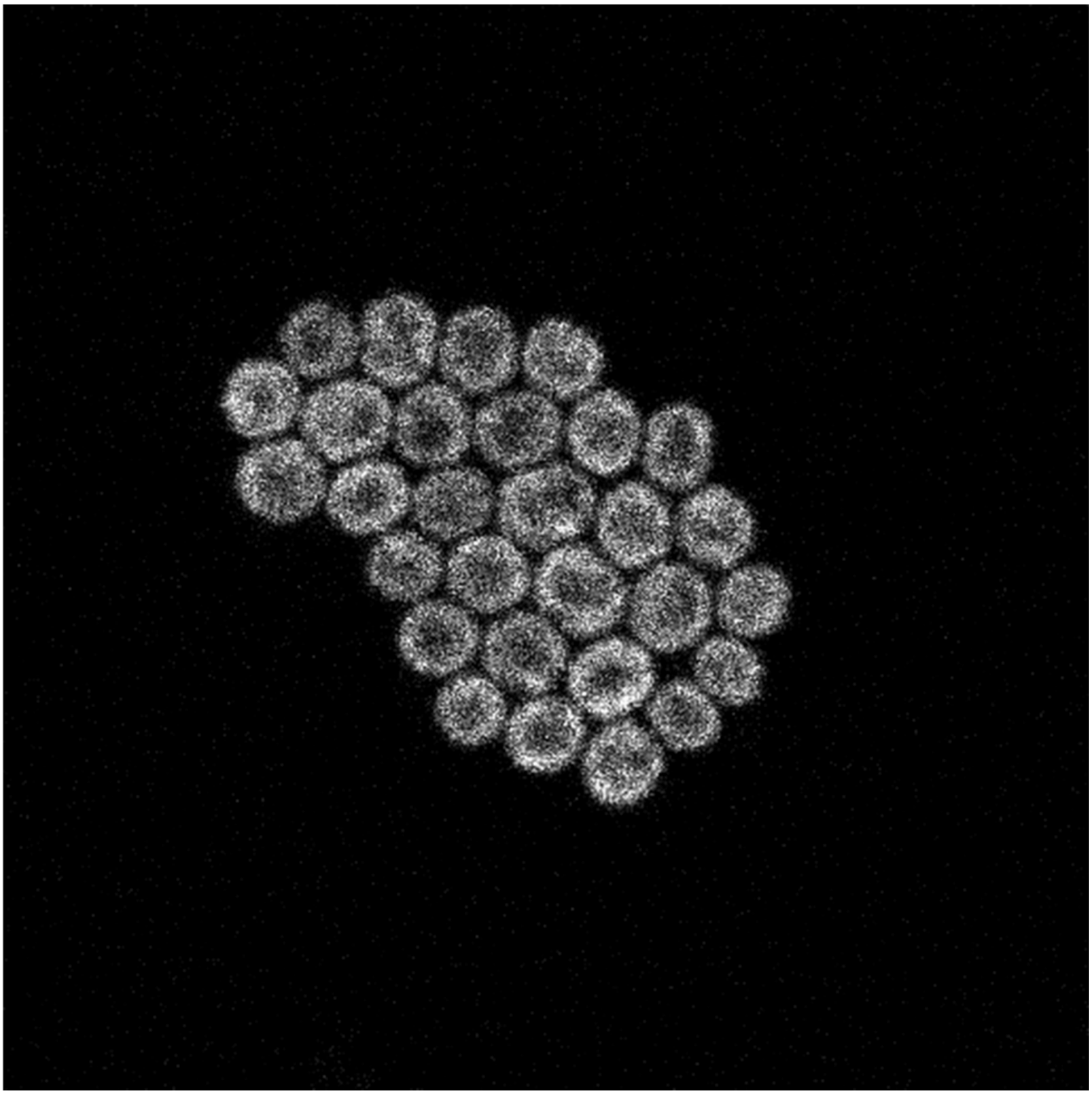

Supplementary Figure 7A. Representative confocal fluorescence images of *hoxF-GFP* in low light (LL).

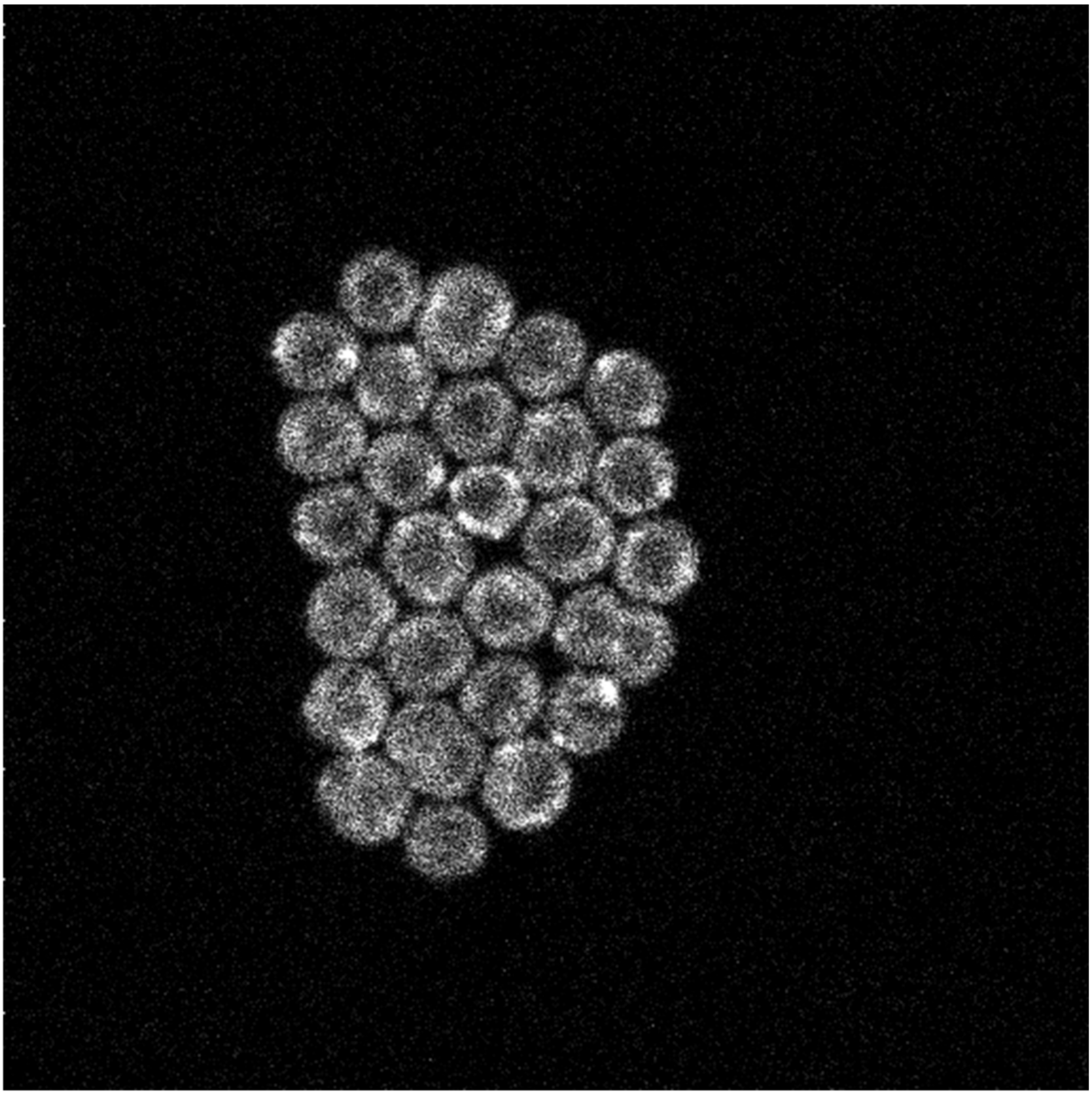

Supplementary Figure 7B. Representative confocal fluorescence images of *hoxF-GFP* in high light (HL).

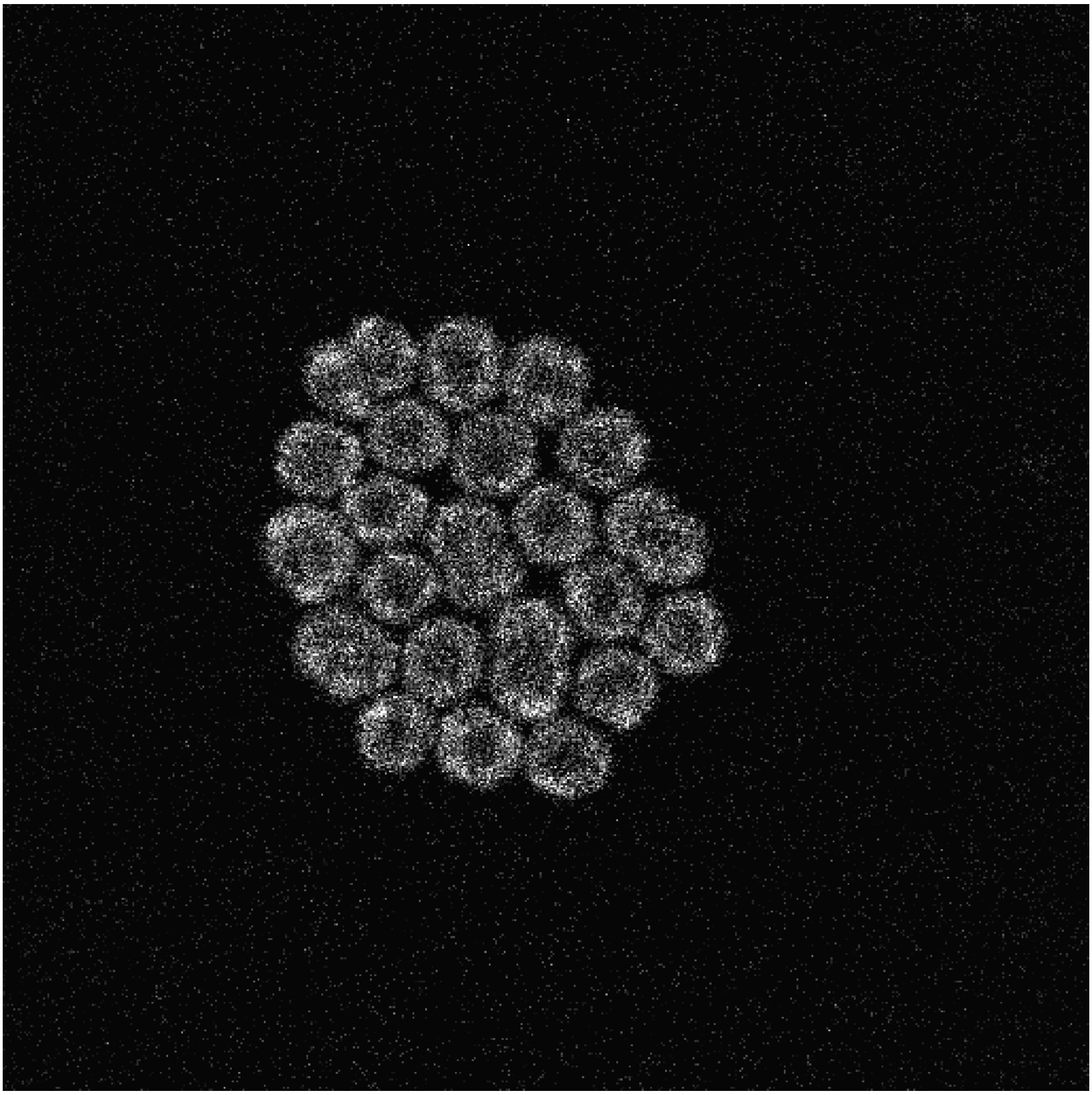

Supplementary Figure 7C. Representative confocal fluorescence images of *hoxF-GFP* following dark adaptation for 3 days.

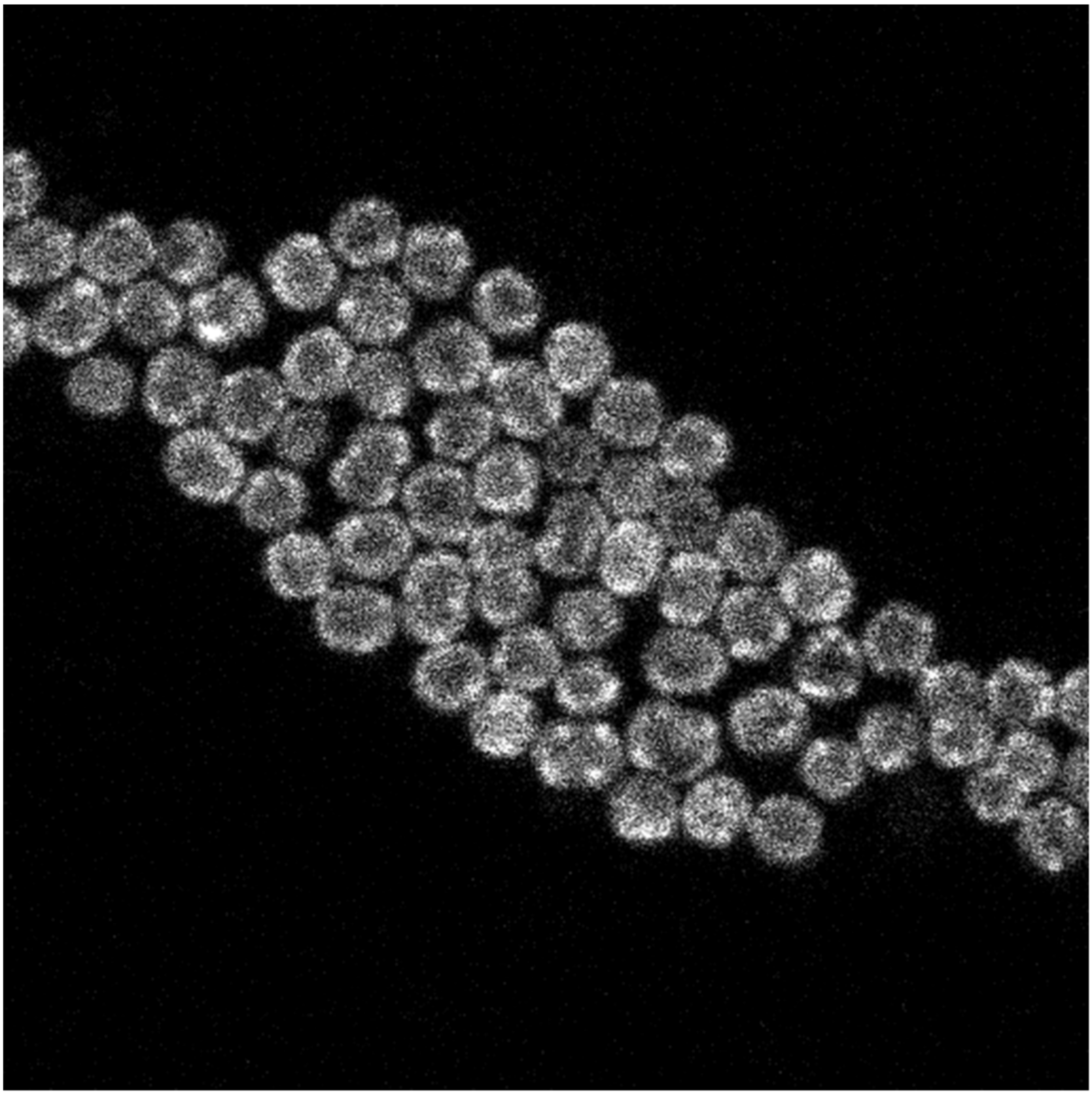

Supplementary Figure 7D. Representative confocal fluorescence images of *hoxF-GFP* following dark adaptation for 5 days (DA).

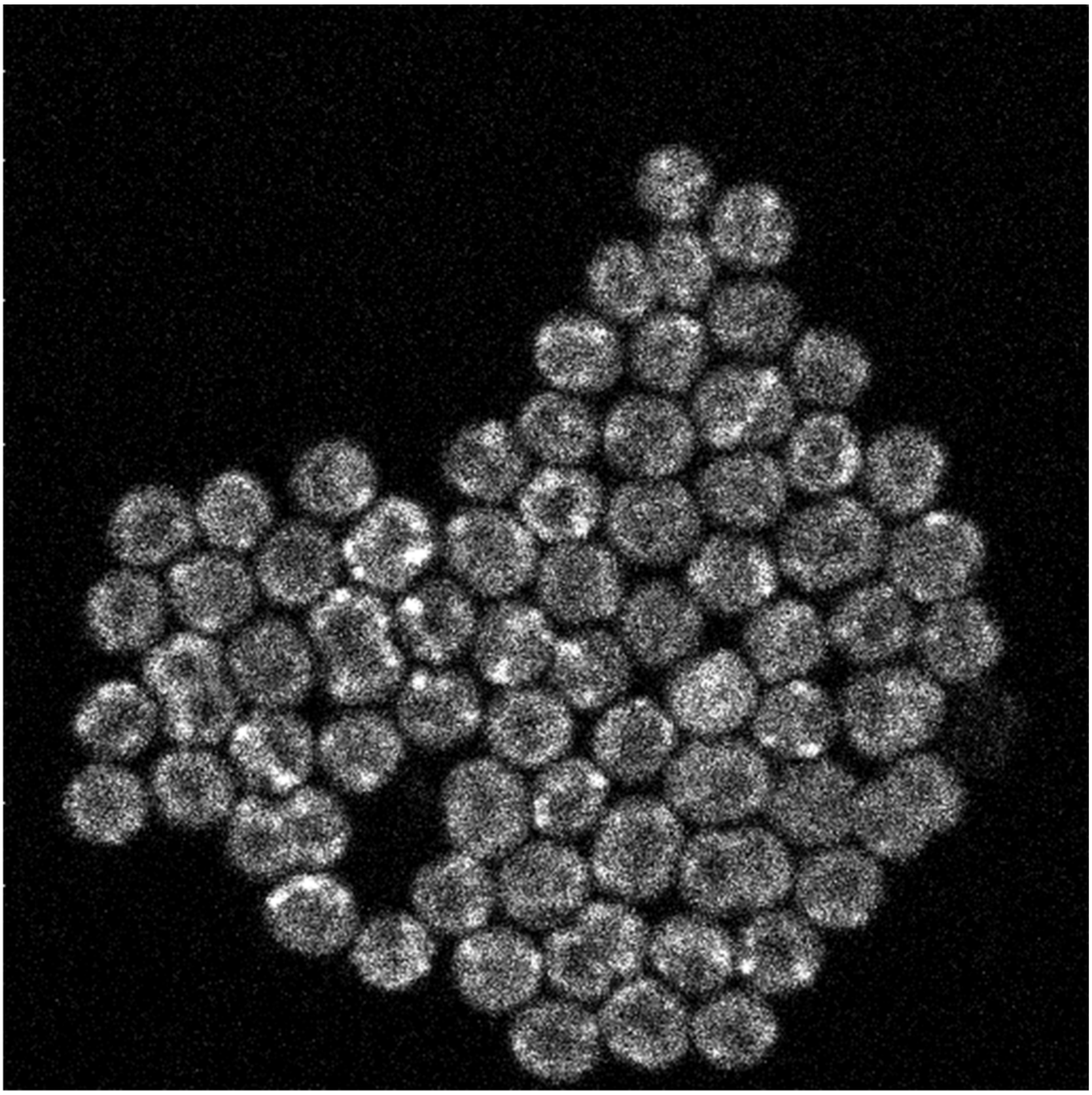

Supplementary Figure 7E. Representative confocal fluorescence images of *hoxF-GFP* following anoxia.

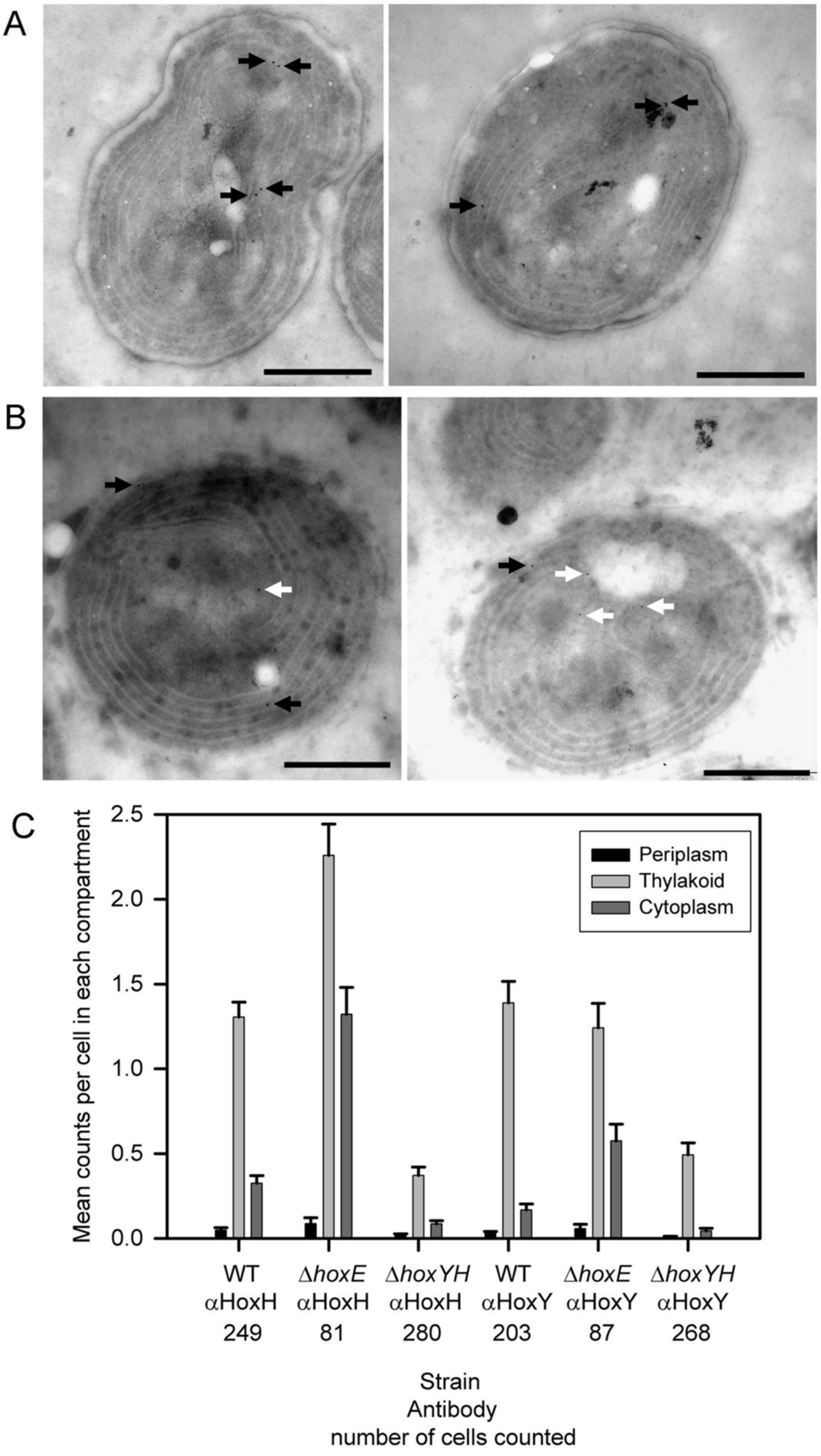

Supplementary Figure 8. Immunogold EM localisation of HoxH and HoxY. Examples of electron micrographs showing immunogold localisation with αHoxY as the primary antibody. Black arrows highlight thylakoid membrane spots, and white arrows highlight cytoplasmic spots. Scale-bars 500 nm. **A**. *Synechocystis* wild-type; **B**. Δ*hoxE*. **C**. Mean

counts per cell (± S.E.) in the indicated strains and cell compartments, with the indicated antibodies.

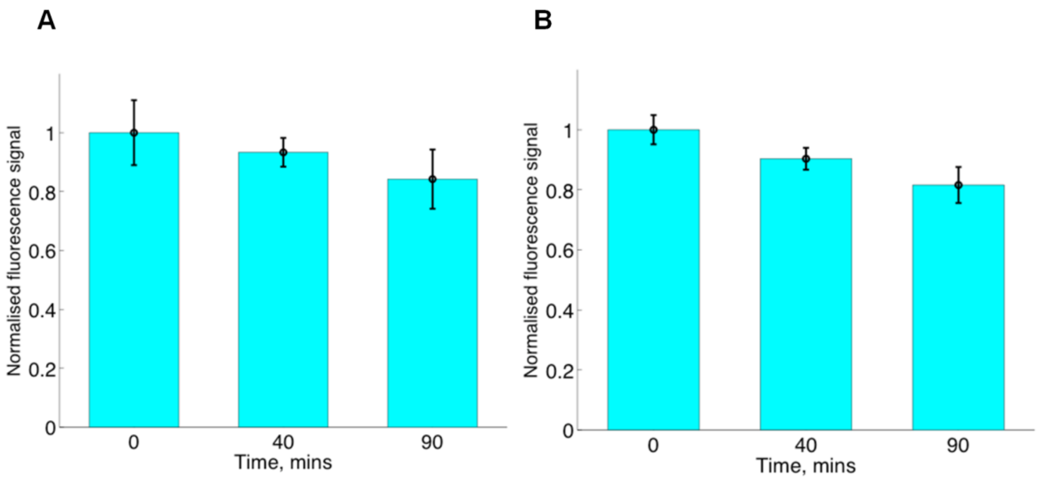

Supplementary Figure 9. **Cell pixel fluorescence with time under (A). Lincomycin treatment (100μg/ml), (B). No treatment.** DA cells are switched to HL at time 0mins with/without addition of Lincomycin at 0 min. Cells are automatically detected in images (see methods) and pixel fluorescence corrected by subtraction of bleached image. Data consists of 6 images per time point (cells from same batch), giving over 600 cells per time point. Mean and standard error are shown at each time point; data normalised (100%) to mean fluorescence at 0 min.

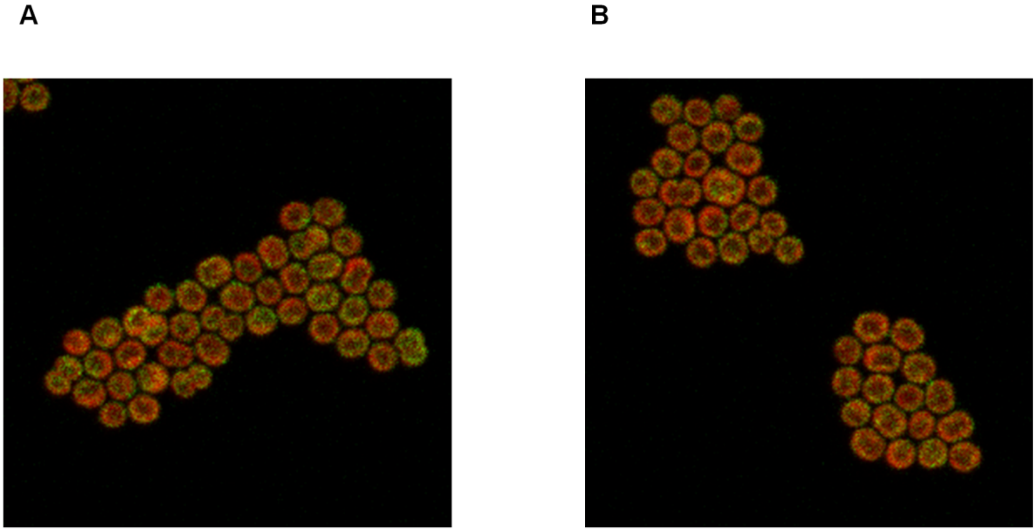

Supplementary Figure 10. Representative confocal images of *hoxF-GFP* following addition of DCMU and DBMIB. (**A**) *hoxF-GFP* cells grown in low light for 1 hr with the addition of 20µM of DCMU. (**B)** *hoxF-GFP* cells grown in low light for 1hr with the addition of 5 µM DBMIB. Overlay images: chlorophyll in red, GFP in green.

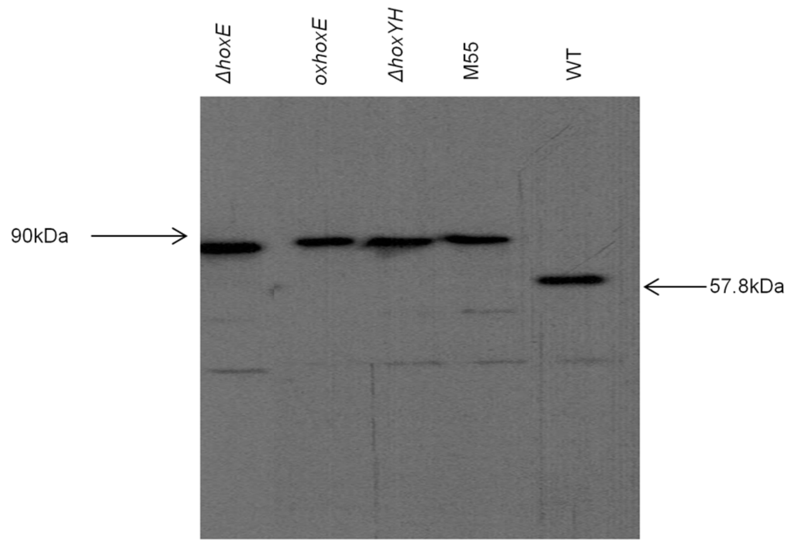

Supplementary Figure 11. Confirmation of complete segregation of HoxF-GFP in Synechocystis strains *ΔhoxE, ΔhoxE* /*hoxE* ox, *ΔhoxHY*, M55 and wild type. Western blot with HoxF antibodies on *ΔhoxE*, *ΔoxHoxE*, *ΔhoxYH*, M55 and wild type whole cell proteins showing complete segregation of HoxF-GFP in each strain.

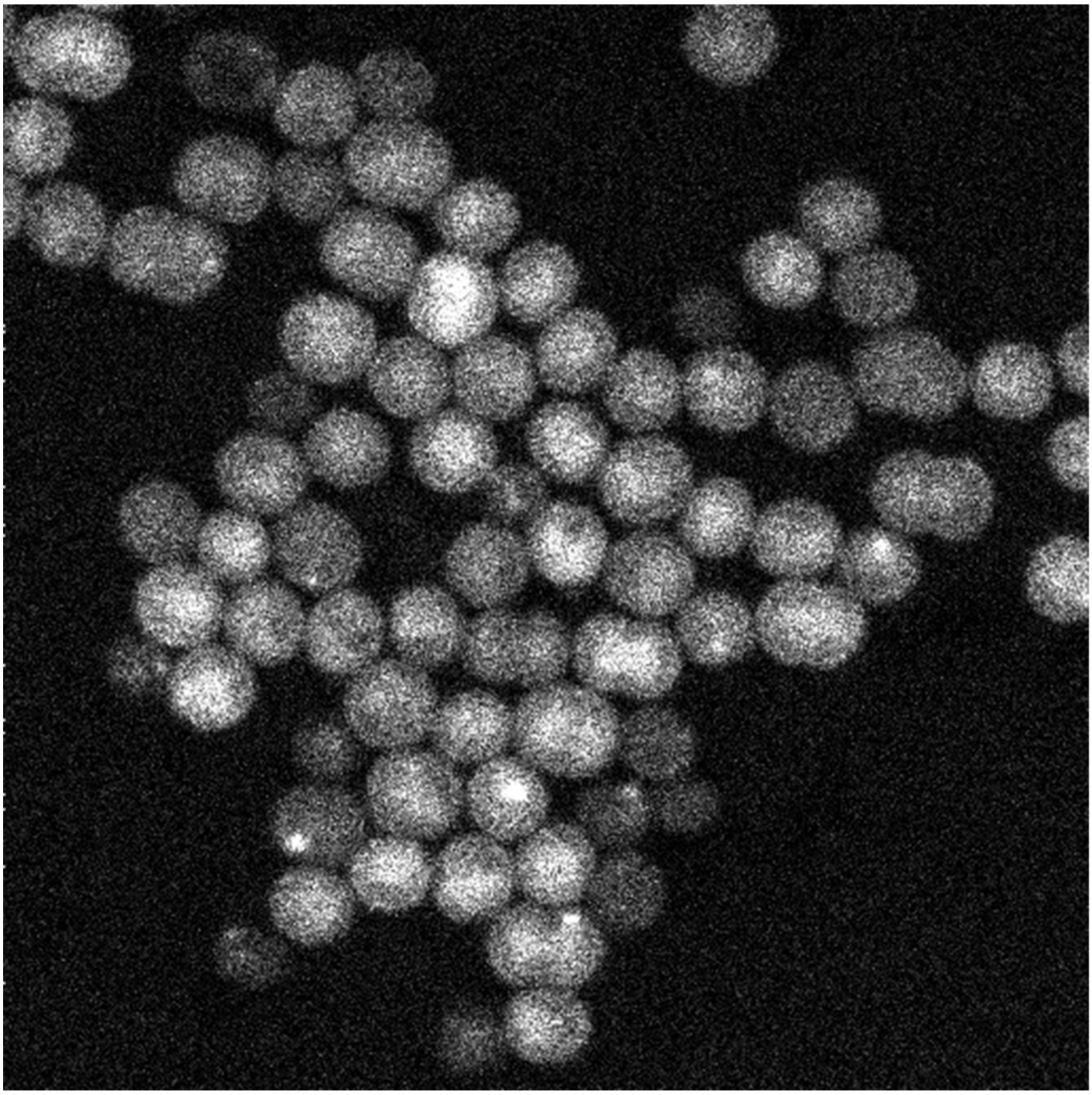

Supplementary Figure 12A. Representative confocal fluorescence image of Δ*hoxE* with HoxF-GFP in low light (LL).

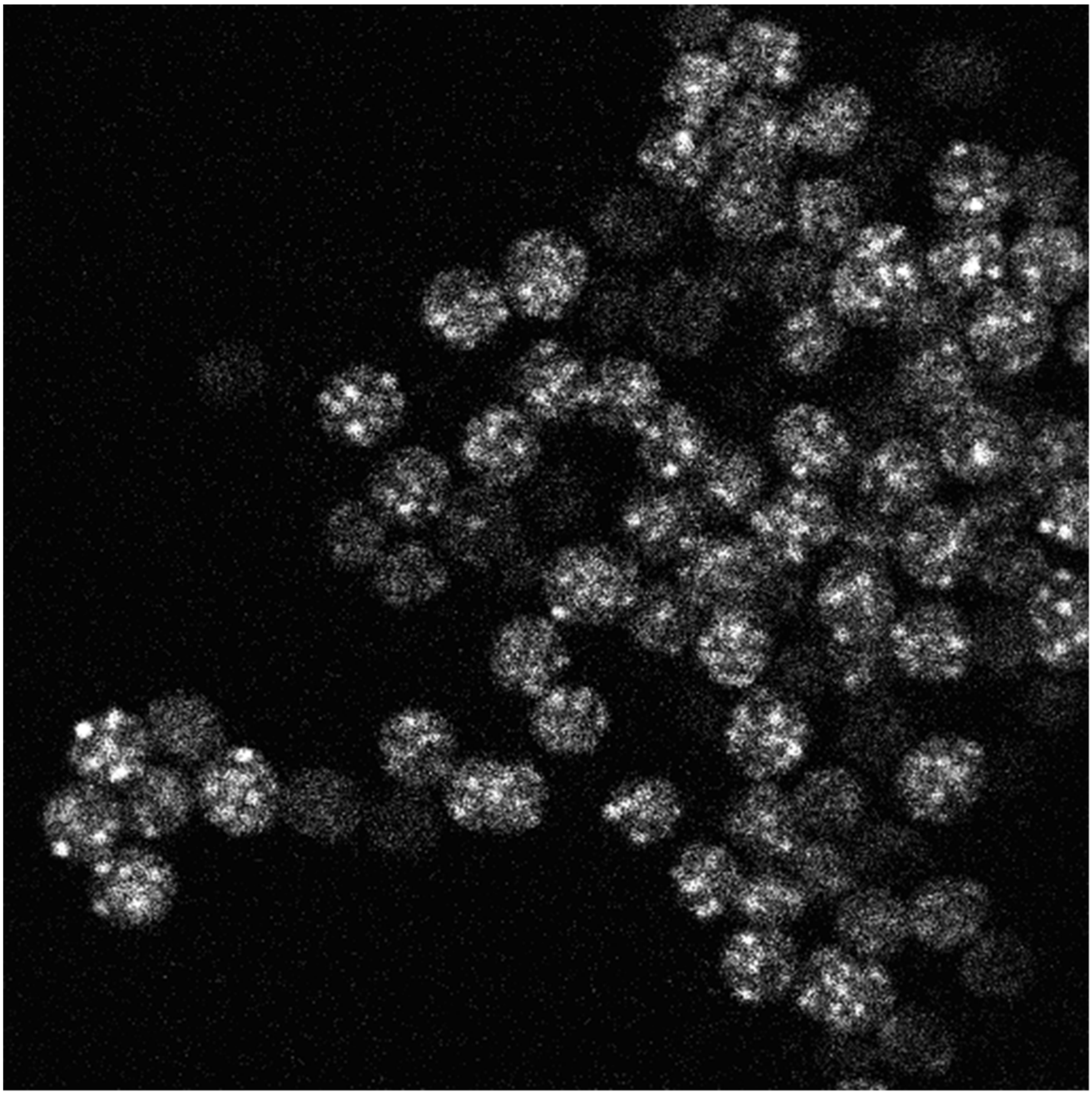

Supplementary Figure 12B. Representative confocal fluorescence image of Δ*hoxE* with HoxF-GFP in high light (HL).

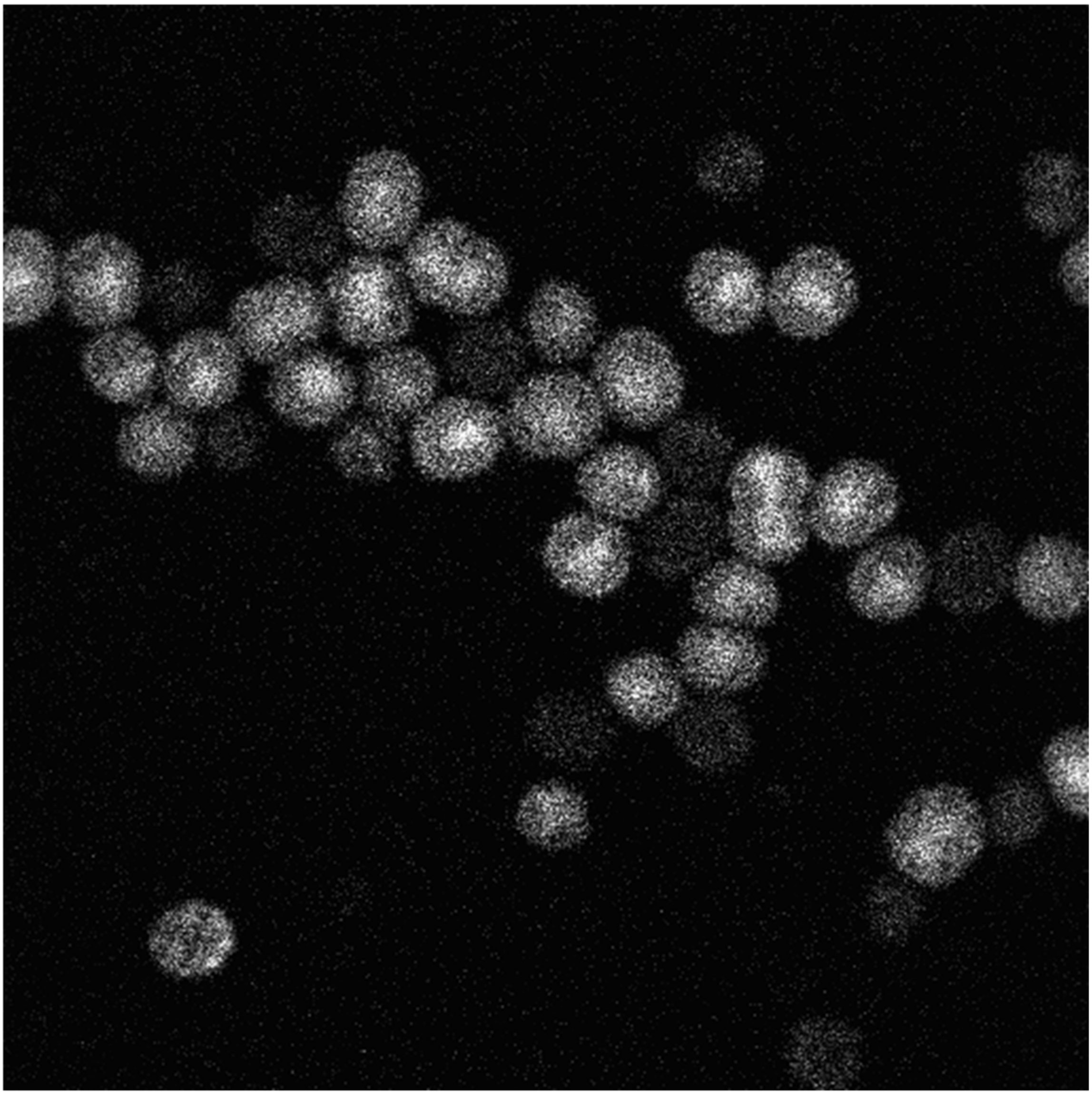

Supplementary Figure 12C. Representative confocal fluorescence image of Δ*hoxE* with HoxF-GFP following dark adaptation (DA) for 3 days.

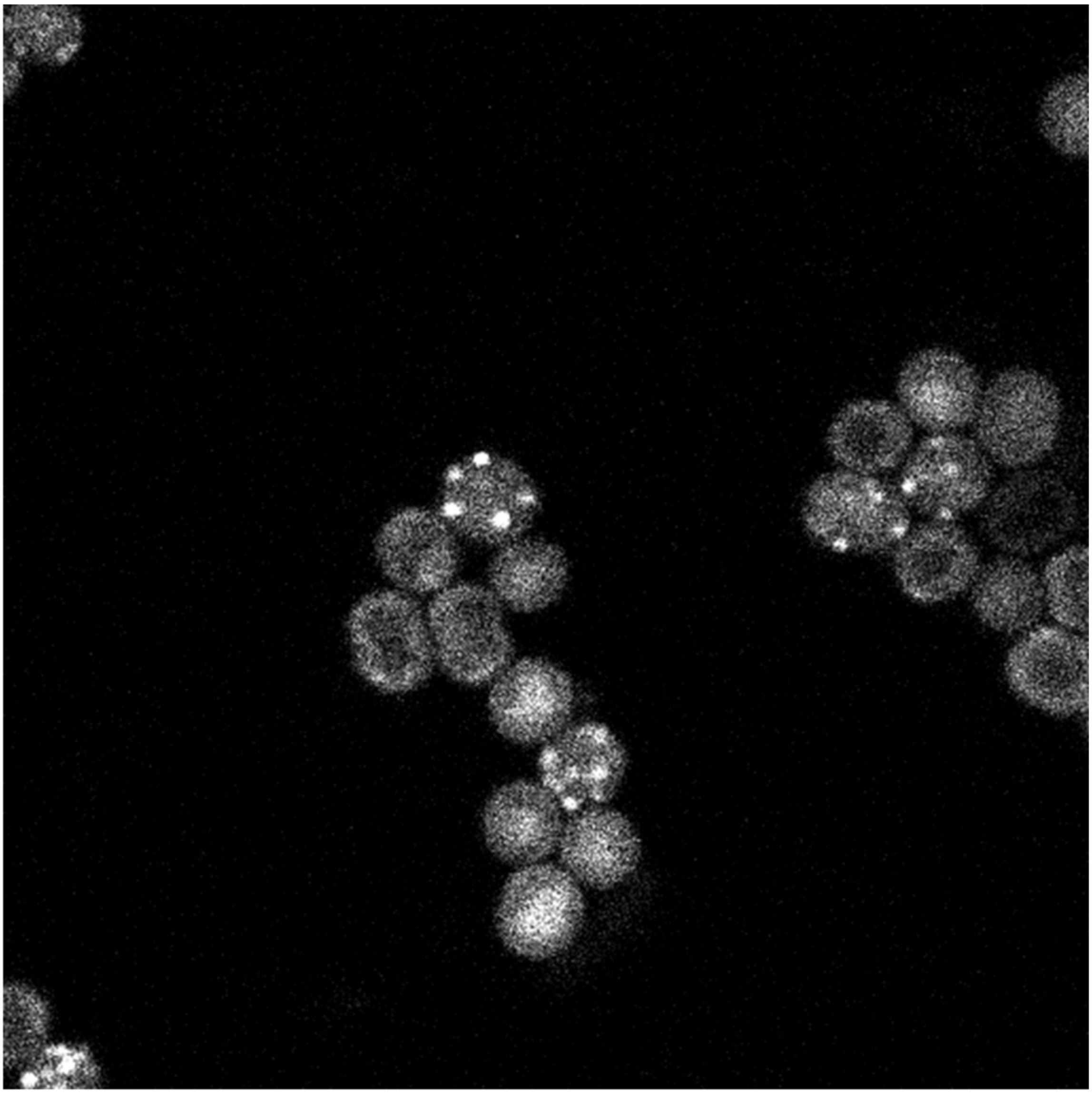

Supplementary Figure 12D. Representative confocal fluorescence image of Δ*hoxE* with HoxF-GFP dark adaptation (DA) for 5 days.

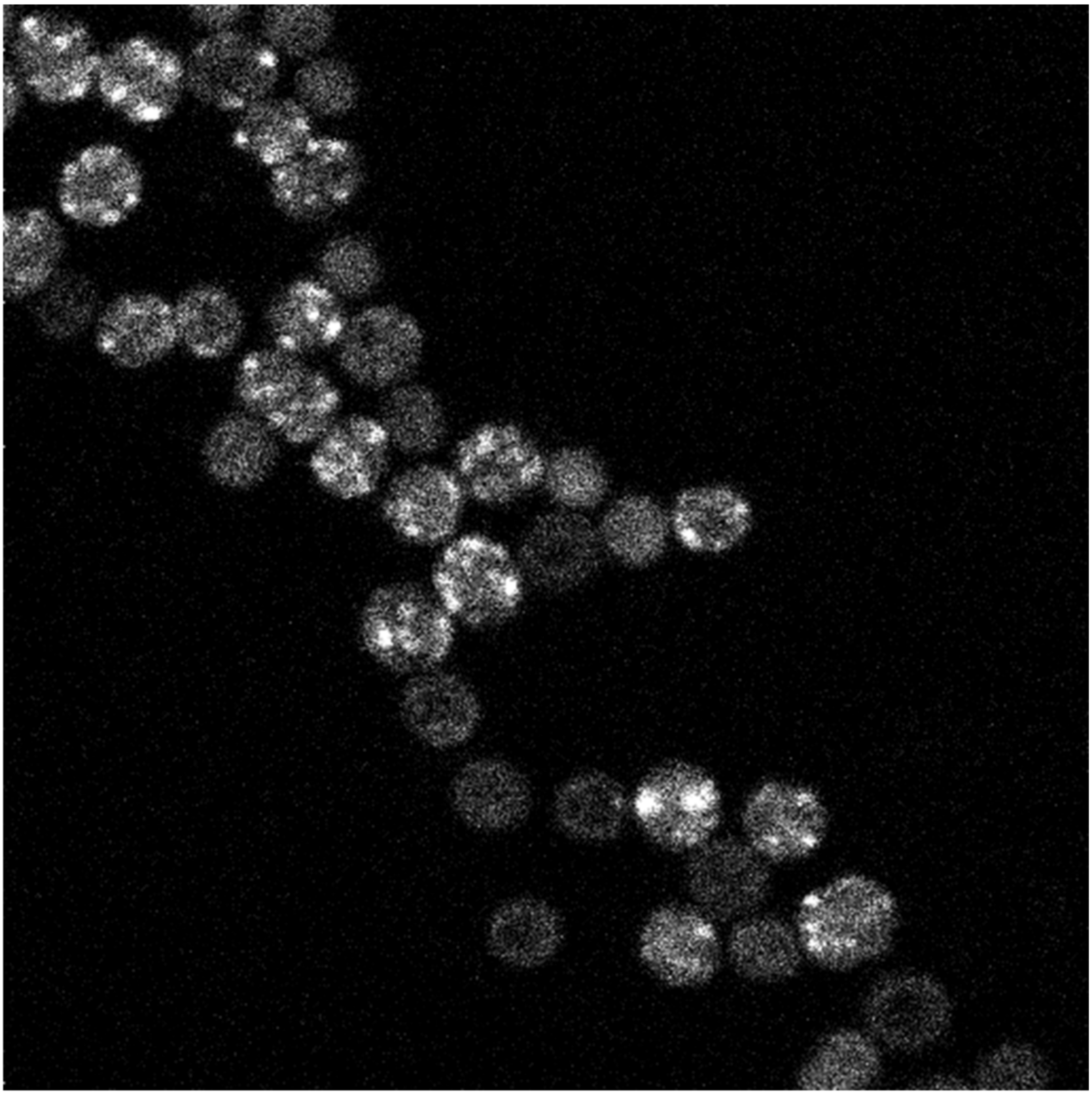

Supplementary Figure 12E. Representative confocal fluorescence image of Δ*hoxE* with HoxF-GFP following anoxia.

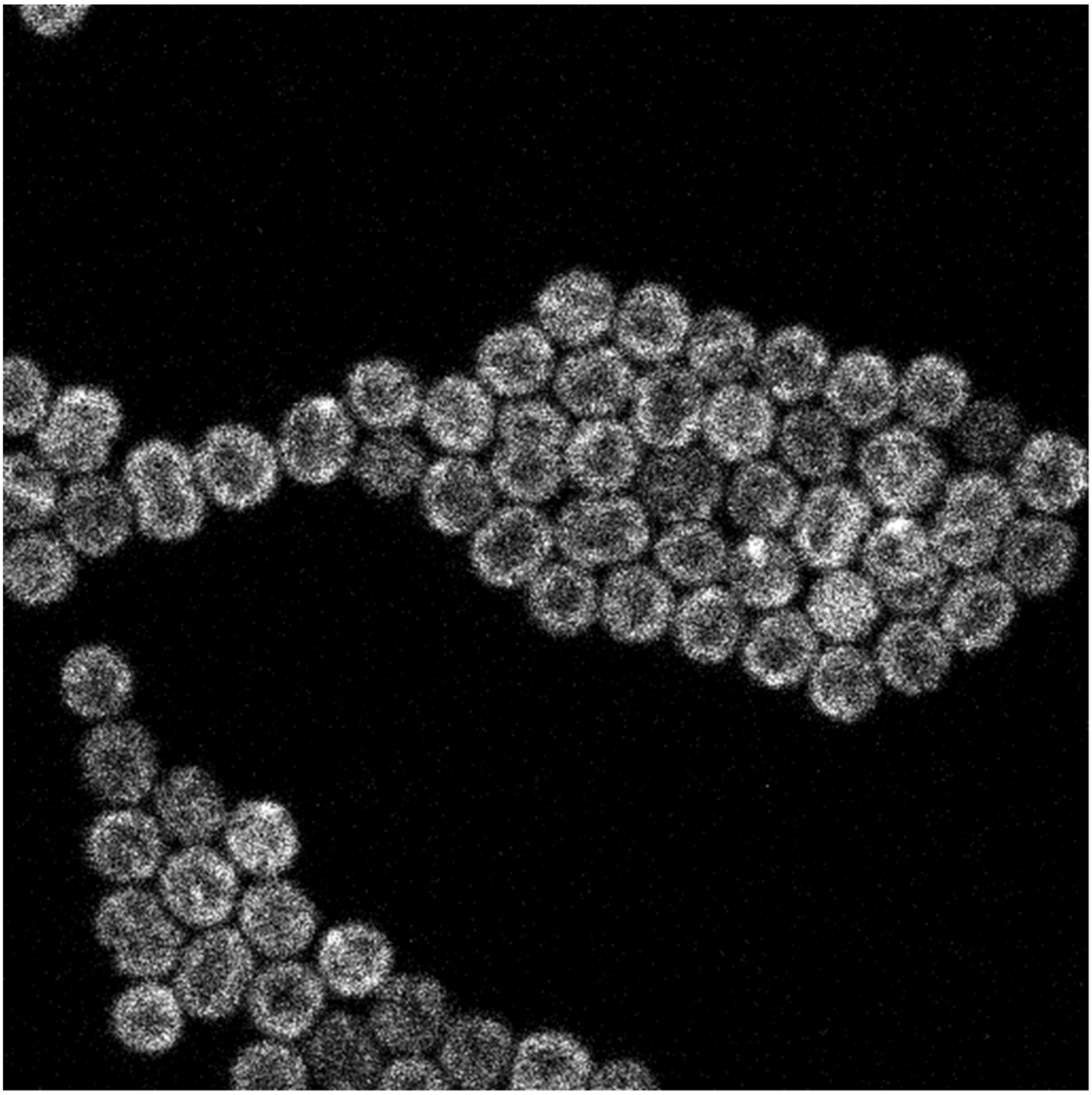

Supplementary Figure 13A. Representative confocal fluorescence image of *oxhoxE* with HoxF-GFP in low light (LL).

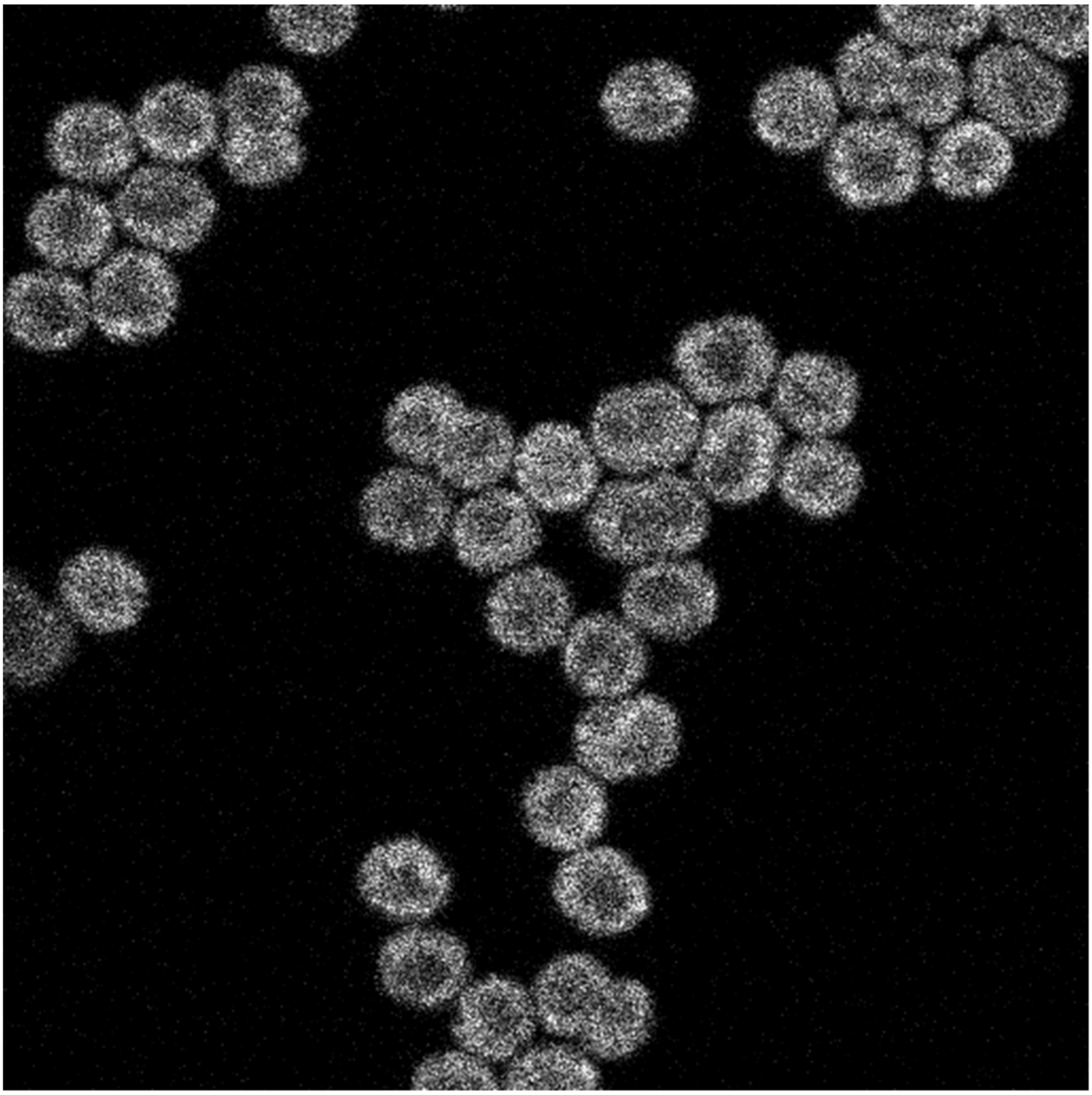

Supplementary Figure 13B. Representative confocal fluorescence image of *oxhoxE* with HoxF-GFP in high light (HL).

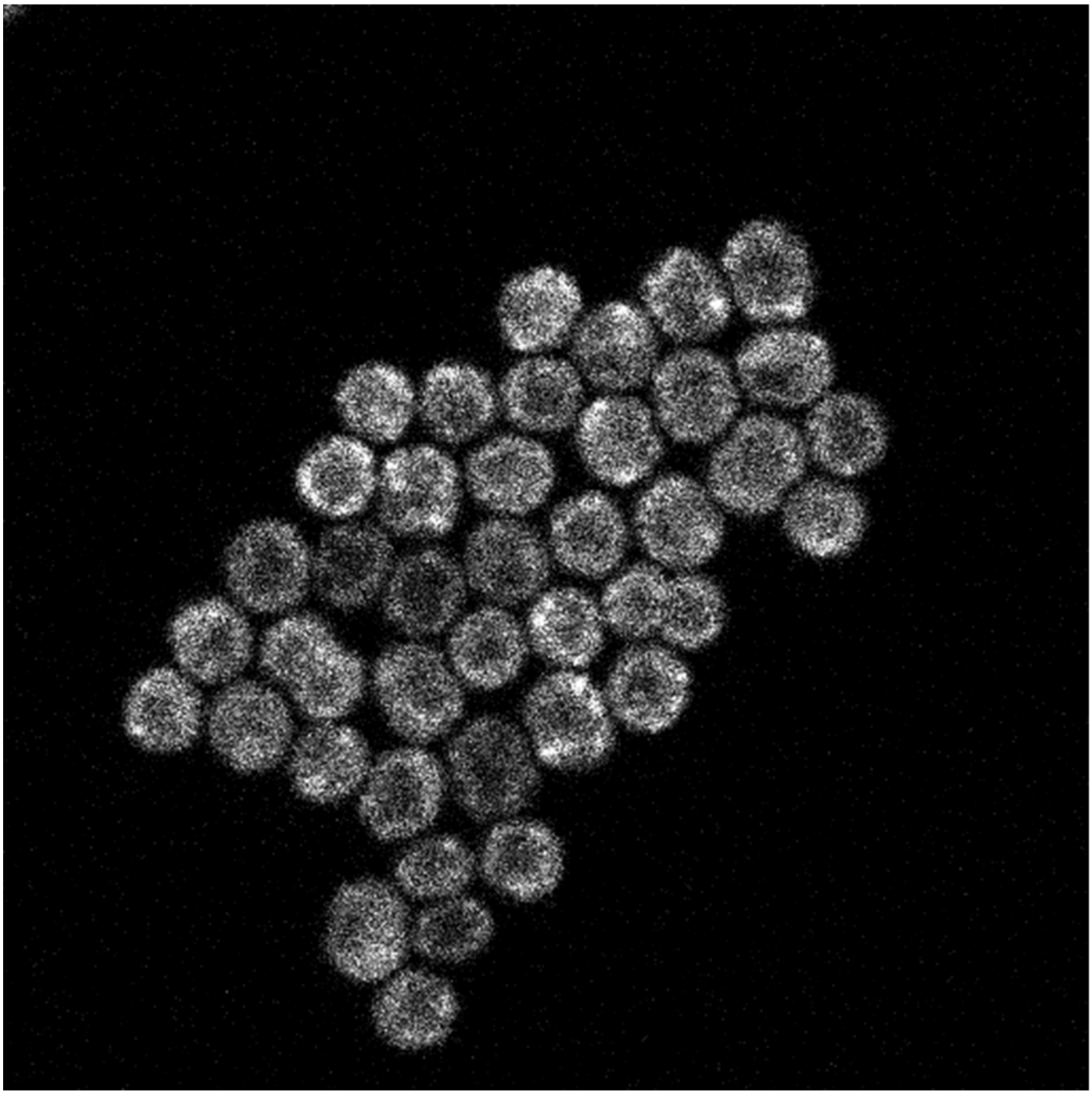

Supplementary Figure 13C. Representative confocal fluorescence image of *oxhoxE* with HoxF-GFP following dark adaptation (DA) for 5 days.

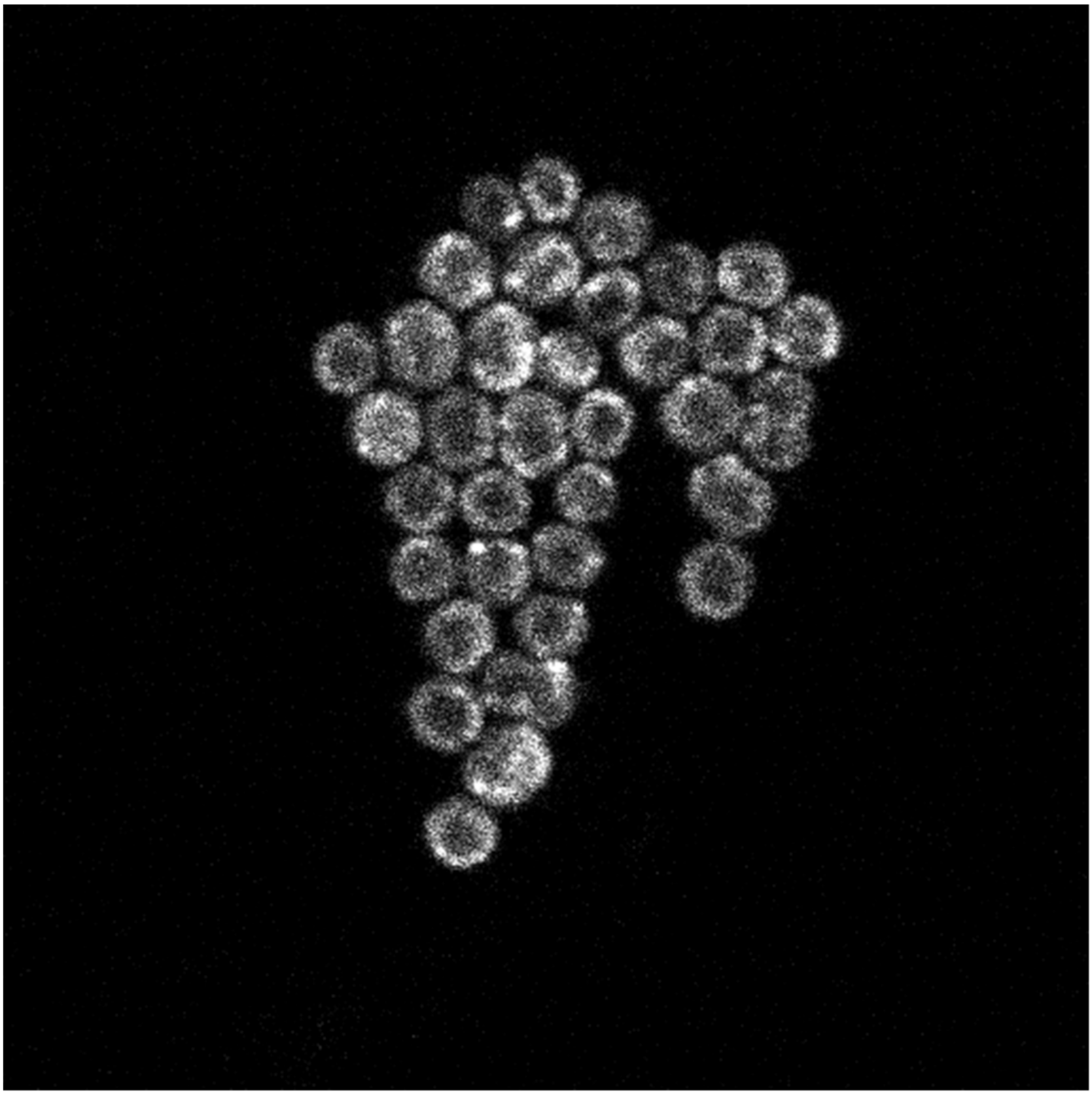

Supplementary Figure 13D. Representative confocal fluorescence image of *oxhoxE* with HoxF-GFP following anoxia.

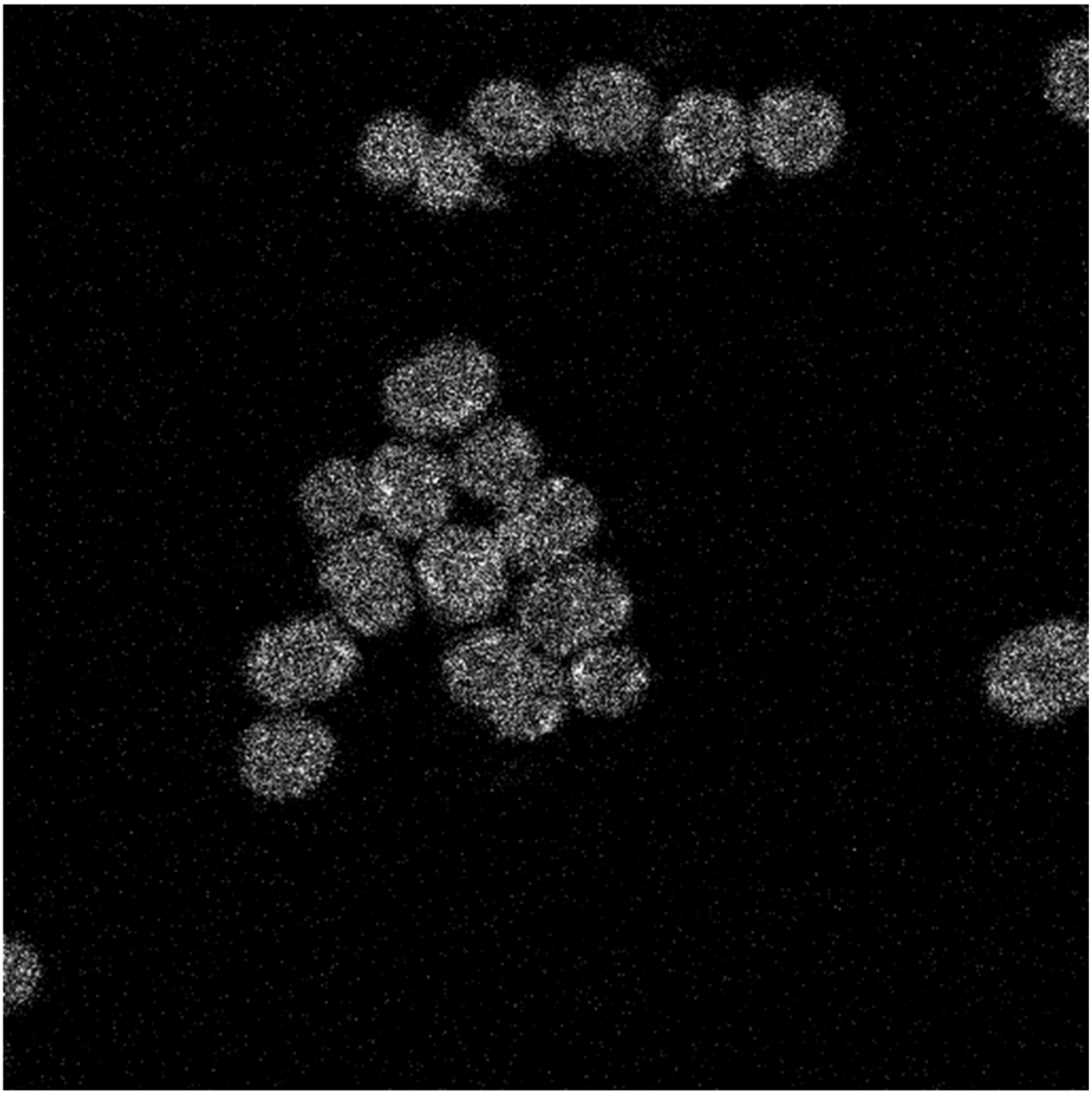

Supplementary Figure 14A. Representative confocal fluorescence image of Δ*hoxYH* with HoxF-GFP in low light (LL). Images are normalised such that the 99th percentile in the intensity histogram is white.

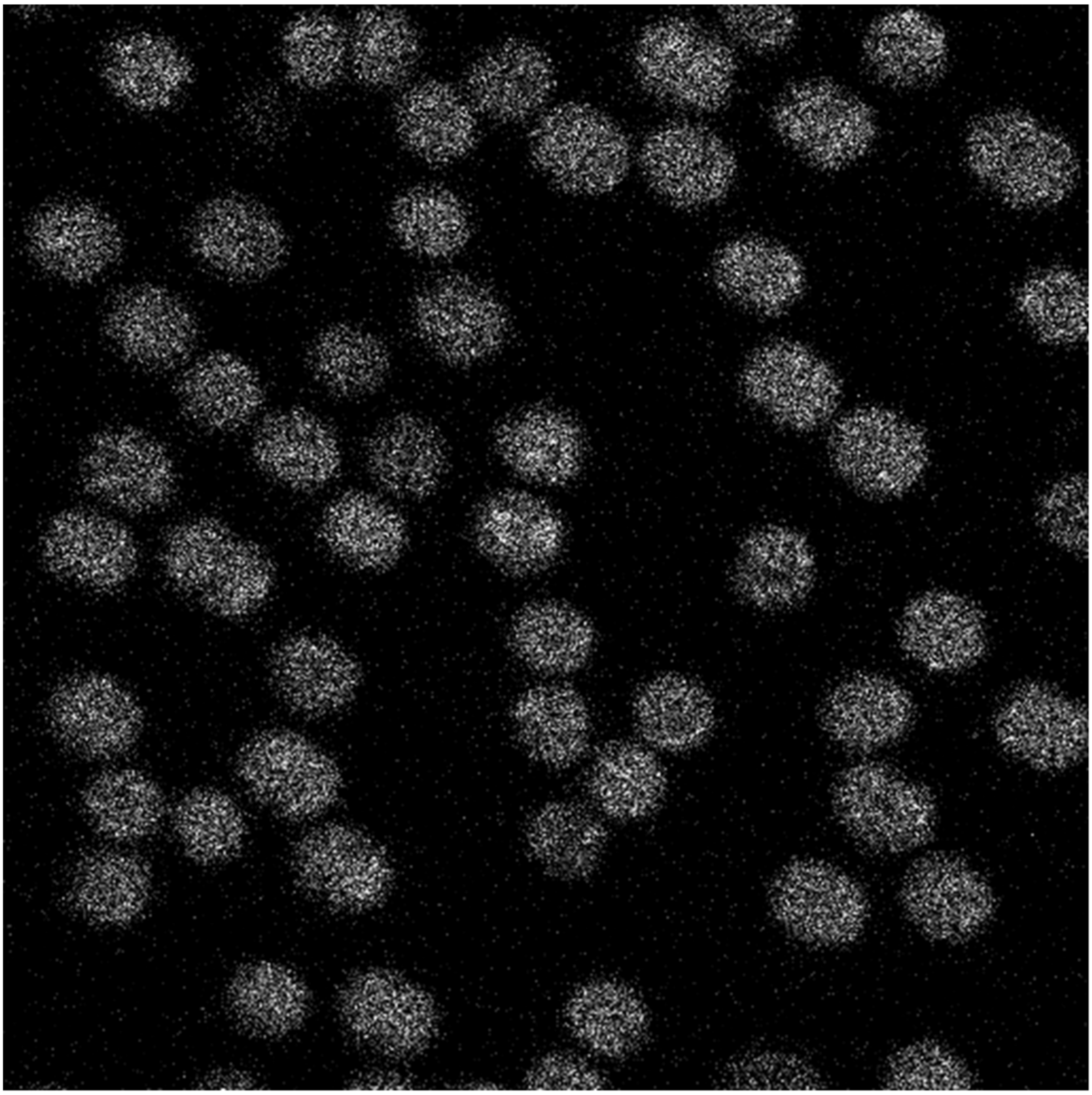

Supplementary Figure 14B. Representative confocal fluorescence image of Δ*hoxYH* with HoxF-GFP in high light (HL). Images are normalised such that the 99th percentile in the intensity histogram is white.

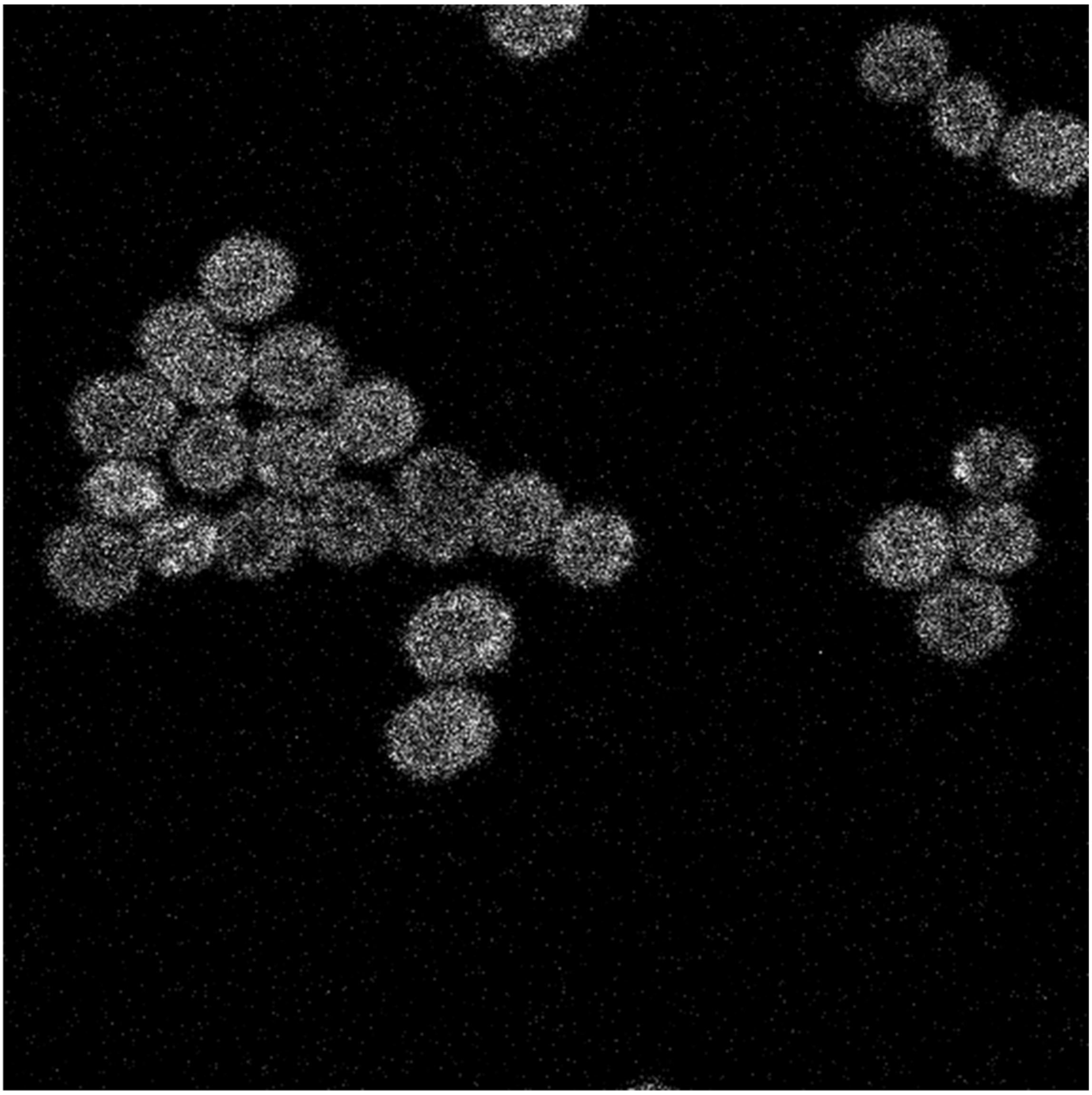

Supplementary Figure 14C. Representative confocal fluorescence image of Δ*hoxYH* with HoxF-GFP following dark adaptation (DA) for 5 days. Images are normalised such that the 99th percentile in the intensity histogram is white.

	nmoles H_2 ml^{-1} min^{-1}	nmoles H_2 ml^{-1} min^{-1}
	1mM Methyl Viologen	1ml dark anaerobic culture
Wild type	0.326±0.03	0.021±0.02
hoxF-GFP	0.261±0.02	0.020±0.04

Supplementary Table 1: Hydrogenase assay comparing WT and *hoxF-GFP.* Comparable levels of hydrogen production were seen between both the wild type and *hoxF-GFP* strains. Assays were performed as described in Eckert *et al*., 2013. Each assay was done in triplicate.
